# Trend and Burden of Vitamin A Deficiency in 1990–2021 and Projection to 2050: A Systematic Analysis for the Global Burden of Disease Study 2021

**DOI:** 10.3390/nu17030572

**Published:** 2025-02-04

**Authors:** Kelly Lin, Yanfei Qi, Jing Sun

**Affiliations:** 1 Rural Health Research Institute, Charles Sturt University, Orange, NSW 2800, Australia; kelly.lin@griffithuni.edu.au; 2 School of Medicine and Dentistry, Griffith University, Gold Coast, QLD 4215, Australia; 3 School of Health Science and Social Work, Griffith University, Gold Coast, QLD 4215, Australia; 4 Centenary Institute, The University of Sydney, Sydney, NSW 2050, Australia; 5 Data Science Institute, University of Technology Sydney, Sydney, NSW 2000, Australia

**Keywords:** vitamin A deficiency, vitamin A supplementation, child malnutrition, VAD

## Abstract

**Background/Objectives**: In this study, we aim to provide an update on the global, regional, and national trends in VAD-associated mortality and morbidity for children under 20 years of age, across different age groups and sociodemographic backgrounds, to identify populations at risk that require further attention. **Methods**: Data from the Global Disease of Burden study were analysed to determine the temporal trends in VAD mortalities and VAD disease burden through disability-adjusted life years (DALYs) and Years Lived with Disability (YLD). Data on children under 20 years of age from 1990 to 2021 from 204 countries and territories were included for analysis. The Average Annual Percentage Change (AAPC) was used to show a temporal trend over a 30-year period. **Results**: Global VAD-associated mortality has decreased significantly, with an AAPC of −0.91 (95% CI= −0.95 to −0.85). No significant improvements in VAD morbidities were identified across Sub-Saharan African regions. In Central Sub-Saharan Africa, the number of VAD-associated disabilities increased from 70,032.12 to 73,534.15. Significant heterogeneity in changes in VAD morbidities were also identified across different countries. The highest age-standardized rate (ASR) of VAD YLD was 282.36 in Somalia, while countries with high sociodemographic indices had an ASR of 0. **Conclusions**: Significant global improvements in VAD mortalities indicate the efficacy of wide-scale high-dose vitamin A supplementation for children under 5 years of age. However, the lack of improvements in VAD morbidities in low-SDI countries highlights the need to continue crucial high-dose vitamin A supplementation and to implement additional vitamin A supplementation programs.

## 1. Introduction

Vitamin A deficiency (VAD) is a critical public health problem in low- and middle-income countries (LMICs) that significantly increases the risk of morbidity and premature mortality for children under 5 years of age [1]. Amongst different micronutrient deficiencies, VAD remains one of the greatest contributors regarding disease burden to the global under-5 mortality rate, serving as one of the four major global nutritional deficiencies [2,3]. Due to the effects of vitamin A on immune function, VAD has been associated with an increased risk in mortality for measles, diarrhoea, and other illnesses in children [4]. Furthermore, VAD is a major cause of xerophthalmia, leading to night blindness and permanent blindness [4]. The global prevalence of VAD among children is estimated to be 29%, with higher rates in Sub-Saharan Africa (49%) and South Asia (44%) [1].

Given the high prevalence of VAD in LMICs and its impacts on child mortality and morbidity, the World Health Organization (WHO) has recommended high-dose vitamin A supplementation for children residing in areas where VAD is a public health problem. The current recommended vitamin A supplementation regime advises 30 mg retinol equivalent doses for children aged 6 to 11 months and 60 mg for those aged 12 to 59 months, administered at least twice a year [5]. The current high-dose vitamin A supplementation programs are focused on enhancing immune function to mitigate major causes of under-5 mortalities. However, the current supplementation regimes are unable to maintain optimal levels of vitamin A in populations with low dietary intake [6,7]. Previous studies have indicated that while high-dose vitamin A supplementation is effective in enhancing immunity to reduce VAD-associated infectious mortalities, improvements in vitamin A status only last for up to 3 months in children with low dietary intake [7]. Thus, ongoing supplementation and additional interventions are necessary to protect against VAD-associated morbidities [4,6,7].

Following the WHO’s recommendations, global initiatives and national policies have been implemented to address VAD. With dedicated global efforts, cause-specific child mortalities associated with VAD have been significantly lowered [1]. In particular, child mortalities pertaining to diarrhoea and measles have decreased worldwide due to significant improvements in VAD treatment regimes [2]. From 1991 to 2013, the global prevalence of VAD decreased from 39% to 29% [1]. However, the prevalence of VAD remains high in Sub-Saharan Africa and South Asia, affecting nearly half of the children in these areas. This highlights the need to maintain large-scale implementation of high-dose vitamin A supplementation while improving daily nutrition for children, as dietary intakes in LMICs remain inadequate [2].

The most recent analysis of global and national trends regarding VAD is based on data collected in 2013 [1]. This highlights the need for an updated analysis on changes in the past 10 years in order to assess the efficacy of current vitamin A supplementation programs and identify nations and regions that require further attention. Previous studies have estimated the prevalence of VAD and VAD-associated mortality [1,2]. In contrast, trends in VAD morbidity have been less explored, with the corresponding studies being limited to night blindness. Although large-scale vitamin A supplementation programs have been implemented in many LMICs, consistent high-dose supplementation for children aged 12 to 59 months is difficult to achieve. Thus, addressing the trend in VAD-associated morbidities is critical to ensure healthy child development. In this study, we aim to provide an update on the global, regional, and national trends in VAD-associated mortality and morbidity for children under 20 years of age across different age groups and SDI backgrounds to identify populations at risk that require further attention.

## 2. Materials and Methods

### 2.1. Data Source

Data from the 2021 GBD study that modelled nonfatal disease burden using Dismod-MR version 2.1 were used for analysis in this study. We analysed VAD mortalities and VAD disease burden through disability-adjusted life years (DALYs) and Years Lived With Disability (YLD). Data on children under 20 years of age from 1990 to 2021 from 204 countries and territories were included for analysis. Countries were further classified into 5 groups based on sociodemographic index (SDI) and 21 GBD regions according to geographical contiguity. Children were further divided by age group. We focused on analysing results for children under 5 years of age as they are most vulnerable to VAD-associated mortalities. Furthermore, WHO-recommended bi-annual high-dose vitamin A supplementation programs are also targeted at children under 5 years of age.

### 2.2. SDI

Sociodemographic index (SDI) is a summary score that measures a country’s social and economic development. The SDI score is calculated based on a nation’s economy as measured by lag-distributed income (LDI) per capita, mean education for those 15 and older, and total under 25 fertility rates of nations. This index has been used in GBD studies, as factors assessed to determine SDI strongly correlate with health outcomes. Countries included in this study are classified into one of five categories based on their SDI scores—high, high-middle, middle, low-middle, and low.

### 2.3. Statistical Analysis

To determine the temporal trend of VAD disease burden and mortality for children and adolescents, the Average Annual Percentage Change (AAPC) was calculated. The AAPC is a suitable summary measure that has been used to determine epidemiological trends in child mortality and morbidity [8,9]. Vitamin-A-deficiency-related DALYs, YLD, and mortality numbers and rates per 100,000 persons across 5 age groups (<5 years, 5 to 9 years, 10 to 14 years, 15 to 19 years, and <20 years) were used to calculate AAPC. For national results, age-standardized rates per 100,000 persons were used for AAPC calculations. The AAPC was used to show a temporal trend over a 30-year period. A positive result indicates an increasing trend in disease burden and mortality, and a negative AAPC indicates a decreasing or improving trend in disease burden and mortality. Level of statistical significance was measured using 95% confidence interval (CI), indicating the stability of the trend.

## 3. Results

### 3.1. Global

**Figure 1:**
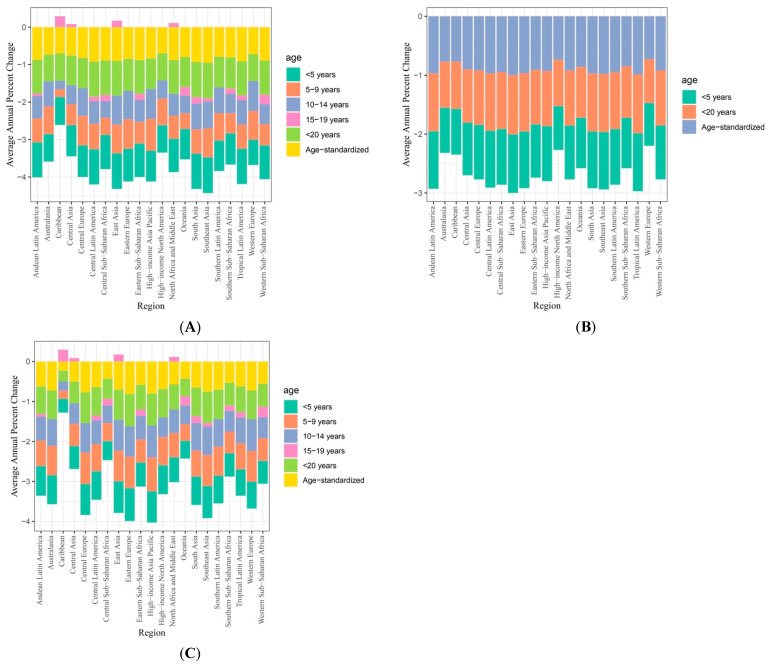
Regional vitamin A AAPC (**A**) DALYs, (**B**) mortalities, and (**C**) YLD from 1990 to 2021.

Significant reductions in VAD mortality, DALYs, and YLD were found from 1990 to 2021 (Figure 1, Table 1). Global under-20 VAD-associated mortality decreased from 207,555.22 to 17,374.40, with an AAPC of −0.91 (95% CI = −0.95 to −0.85). Similar reductions in under-20 VAD YLDs were identified, with a reduction from 1,153,473.83 in 1990 to 508,371.76 in 2021, with an AAPC of −0.58 (95% CI = −0.66 to −0.35). However, at older ages, VAD YLD increased from 24,647.31 to 24,613.78, with an AAPC of 0.34 (95% CI = 0.21 to 0.46).

### 3.2. Sociodemographic Index

When countries were divided based on their SDI scores, the most significant improvement in under-20 VAD mortality was found in high-middle SDI countries, for which this figure decreased from 2009.88 to 14.95, with an AAPC = −0.99 (95% CI = −1 to −0.98). The levels of improvement in VAD mortalities were identical in high (AAPC = −0.97, 95% CI = −0.99 to −0.89), middle (AAPC = −0.97, 95% CI = −0.99 to −0.96), and low-middle (AAPC = −0.97, 95% CI = −0.99 to −0.94) SDI countries. Significant disparities in the number of VAD-associated mortalities were identified across high- to low-SDI countries, with a more than 10-fold difference across countries with different SDI levels. The number of VAD-associated deaths was 1.14 for high-SDI countries, 14.95 for high-middle-SDI countries, 454.33 for middle-SDI countries, 2604.91 for high-middle-SDI countries, and 14,285.59 for low-SDI countries.

However, the results differed for disease burden. The greatest improvement in under-5 VAD YLD was identified in high-SDI countries, with a decrease from 3037.75 to 665.1 (AAPC = −0.84, 95% CI = −0.88 to −0.80). Similar levels of improvement were found across high-middle (AAPC = −0.75, 95% CI = −0.82 to −0.66), middle- (AAPC = −0.78, 95% CI = −0.83 to −0.64), and low-middle (AAPC = −0.71, 95% CI = −0.77 to −0.56)-SDI countries. In contrast, although the number of under-20 VAD-associated disabilities decreased in countries with low SDI scores from 656,073.38 to 555,757.69, the AAPC calculations showed no significant improvements in VAD-associated disabilities (AAPC = −0.26, 95% CI = −0.4 to 0.22).

### 3.3. Regional

**Figure 2:**
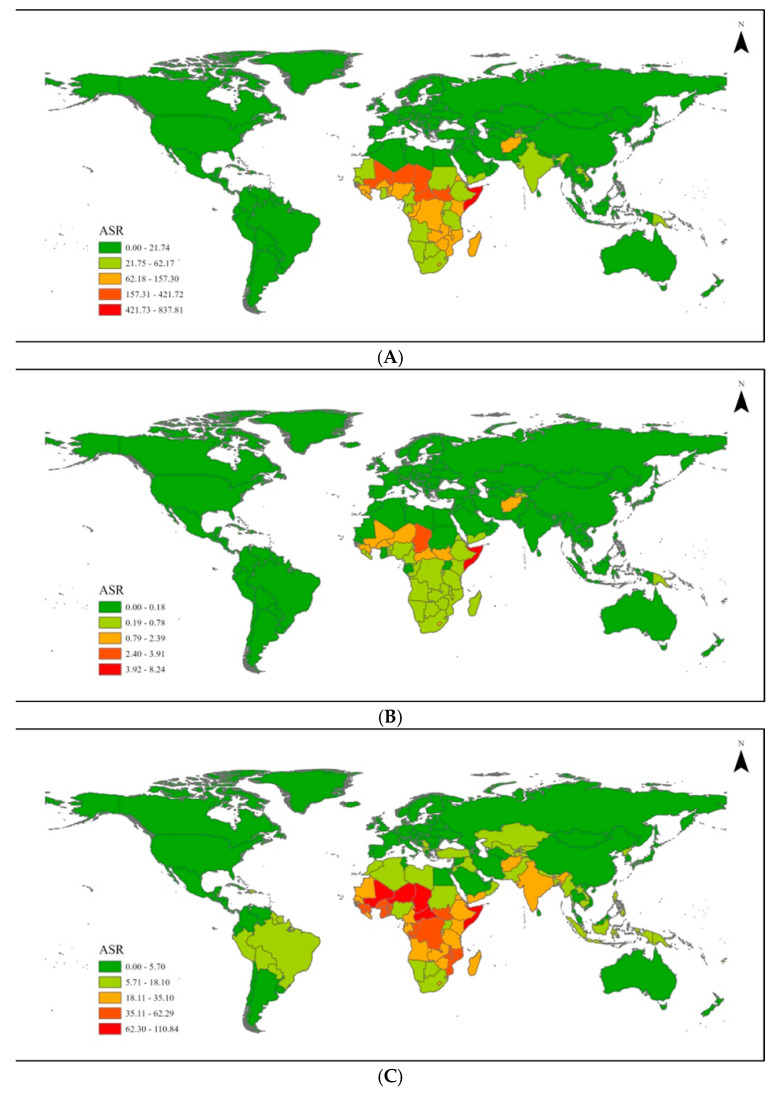
Age-standardized rate of global (**A**) DALYs, (**B**) mortalities, and (**C**) YLD for vitamin A deficiency.

Geographical-region trends in VAD are presented in Table 2 and Figure 2. All geographical regions showed significant improvements in the number and rate of VAD-associated mortalities for children under 5 and 20 years of age. Latin American regions, Southeast Asia, and East Asia showed the most significant improvement in VAD mortalities from 1990 to 2021. In the Latin American regions, the number of VAD-associated mortalities decreased from 1217.69 to 48.44 in Central Latin America (AAPC = −0.97, 95% CI = −0.99 to −0.97) and 261.44 to 8.12 in Andean Latin America (AAPC = −0.97, 95% CI = −0.98 to −0.96).

Although significant improvements were made from 1990 to 2021, the number of VAD-associated mortalities remained the highest in Sub-Saharan African regions and South Asia. In Eastern Sub-Saharan Africa, the number of VAD-associated mortalities decreased from 42,015.64 to 5994.17, with an AAPC of −0.84 (95% CI = −0.94 to −0.71). In Western Sub-Saharan Africa, the number of VAD-associated mortalities decreased from 50,678.07 to 6995.06, with an AAPC of −0.83 (95% CI = −0.96 to −0.62). In South Asia, the number of VAD-associated mortalities decreased from 64,647.38 to 1822.22 (AAPC = −0.97, 95% CI = −1 to −0.95).

Compared to those for VAD-associated mortalities, improvements were more modest in the burden of VAD as measured by YLD. In some age groups and regions, increased burdens of VAD-associated disabilities were identified. Although fewer VAD-associated disabilities were identified in the Caribbean region, i.e., from 6893.2 in 1990 to 6021.77 in 2021, the AAPC (−0.27) did not reach the level of statistical significance (95% CI = −0.5 to 0.12). Similarly, no significant improvements in VAD-associated disabilities were identified in Sub-Saharan African regions, as the AAPCs calculated for Central, Western, and Eastern Sub-Saharan African regions did not reach statistical significance (*p* > 0.05). Furthermore, the burden of VAD-associated disabilities remained the highest in Sub-Saharan Africa regions in 2021. In Central Sub-Saharan Africa, the number of VAD-associated disabilities increased from 70,032.12 to 73,534.15, with an AAPC of 0.19 (95% CI = −0.09 to 0.76). In Western Sub-Saharan African regions, the number of VAD-associated disabilities increased from 243,082.37 to 244,619.34 (AAPC= 0.04, 95% CI = −0.12 to 0.42). In Eastern Sub-Saharan Africa, the number of VAD YLD decreased from 232,549.3 to 188,729.33 (AAPC = −0.23, 95% CI = −0.37 to 0.06). 

### 3.4. National

Unlike the global and regional results, the national VAD results were age-standardized and included both children and adolescents under 20 years of age (Figure 2, Table 3). Similar to the regional results, countries in the Caribbean and Sub-Saharan had higher values.

The African region had the highest age-standardized rate of VAD-associated mortalities and morbidities in 2021. The nations with the highest age-standardized VAD mortality rates per 100,000 children in 2021 were Burkina Faso (3.16), Central African Republic (4.09), Chad (10.41), Guinea-Bissau (4.14), Mali (5.22), Somalia (21.72), South Sudan (6.51), and Zimbabwe (3.24). In contrast, high-income countries in North America and Europe had rates close to or equal to 0, including Romania (0.02), Italy (0), Hungary (0.03), France (0), Sweden (0), and the United States of America (0).

Similarly, the highest age-standardized rates of VAD YLD in 2021 for children under 20 were also identified in Sub-Saharan African and Caribbean regions. Age-standardized rates were calculated per 100,000 children under 20 years of age. The highest age-standardized rate of VAD YLD was recorded in countries such as Benin (120.89), Burkina Faso (134.74), Central African Republic (167.17), Chad (190.30), Gambia (114.60), Guinea (116.70), Niger (245.18), Somalia (282.36), and South Sudan (131.13).

## 4. Discussion

This study reveals significant improvements in VAD-associated mortality across countries of all levels of SDI from 1990 to 2021. Among them, high- to low-middle-SDI countries had more pronounced improvements compared to countries with low SDI scores. Furthermore, countries with low SDI scores showed no significant improvement in VAD-associated disabilities, as measured by YLD. The differences in the improvements in VAD-associated disabilities across different regions further indicate inequalities in malnutrition. While countries in the Latin America and Southeast Asia region showed significant improvements in VAD-associated mortalities and disabilities, countries in Sub-Saharan Africa and the Caribbean region showed no significant improvements in VAD burden.

The observed disparities in improvements among countries of varying SDI scores align with global inequalities in VAD prevalence and vitamin A supplementation. Previous meta-analyses have identified a significantly higher prevalence of VAD in low-SDI regions, while 41.21% of LMICs were classified as areas of moderate to severe VAD public health significance [10]. To address deficiencies in LMICs, vitamin A supplementation has been recommended and implemented globally by the WHO. Improvements in VAD-associated mortality support findings from previous studies that indicated the need to continue vitamin A supplementation and called for more research before the phase-out of vitamin A supplementation programs [11,12]. Previous meta-analyses have identified wide-scale high-dose vitamin A supplementation programs as a major contributor to large reductions in mortality, morbidity, and vision problems in LMICs [12]. Malnutrition due to a low intake of eggs, dairy, or other animal products exacerbate the risk of VAD in children from LMICs [13]. The chronic VAD in these populations suggests that vitamin A supplementation is crucial to avoid mortalities associated with VAD and lower the risk of infection while reducing the severity of infection, improving both morbidity and mortality [2].

The disproportionately higher prevalence of VAD further contributes to the greater burden of associated morbidities in low-income and low-SDI regions [10]. This study has identified significant reductions in VAD mortalities in low-SDI regions, with a lack of improvement in VAD morbidity and associated disabilities. Similarly, on a national level, the highest age-standardized rates of VAD-associated mortality and disability were identified in low-income countries within the Sub-Saharan Africa and Caribbean regions. Although significant improvements in VAD mortalities have been identified in low-SDI regions, the number of deaths remained much higher, at 14,285.59, compared to countries with high (1.14), high-middle (14.95), middle (454.33), and low-middle (2604.91) SDI scores. The significant disparity in VAD-associated mortalities indicates inequalities in terms of malnutrition and the importance of ensuring optimal and sustained vitamin A supplementation for children in low-SDI countries to avoid premature deaths associated with VAD. Currently, the WHO recommends bi-annual vitamin A supplementation for all children under 5 in VAD-endemic regions. However, the coverage of vitamin A supplementation remains suboptimal [14]. This fact aligns with the results of the current study, wherein we found there was a high number of VAD-associated mortalities in low-SDI countries in 2021, suggesting that optimal and sustained vitamin A supplementation is difficult to achieve in these countries, a result consistent with previous findings [10]. When countries are grouped based on geographical regions, countries in the Sub-Saharan Africa and the Caribbean region appeared to have the greatest age-standardized rates of VAD mortality and disability. This is supported by previous studies that have found suboptimal routine delivery (<50%) for vitamin A supplementation in Sub-Saharan African countries, where vitamin A supplementation programs have not achieved the level of coverage (80%) required for a significant public health impact [15]. Due to the chronic malnutrition in low-SDI countries, vitamin A supplementation may act as a life-saving intervention that can help mitigate VAD-associated mortality for children under 5 years of age with conditions such as diarrhoea and measles [6]. More importantly, malabsorption or loss of vitamin A due to disease in LMICs with poor sanitation and endemic infectious diseases further contribute to the higher burden of VAD-associated mortalities [16]. Thus, a multifaceted approach is required to improve sanitation and vitamin A supplementation coverage.

A greater focus on nation-specific risk factors for VAD and obstacles in vitamin A supplementation is also required to address the significant heterogeneity in the improvements in national VAD mortality and morbidity to achieve high coverage of vitamin A supplementation. One of the nation-specific barriers identified in previous studies is the circulation of false vitamin A capsules with insufficient doses within Sub-Saharan Africa regions [17,18]. In the Caribbean region, previous studies have identified difficulties in reaching disadvantaged and vulnerable groups as the main reason for the high levels of VAD observed [19]. Across both Sub-Saharan Africa and the Caribbean, problems regarding the storage of vitamin A and the lack of access to medical services for treating infections have been identified as contributing factors for VAD and poor vitamin A supplementation coverage [16,18,19]. The adequate storage of vitamin A drugs away from heat is critical to improve drug half-lives and ensure optimal vitamin A supplementation [20].

In addition to using high-dose vitamin A supplementation to reduce VAD-associated premature mortality, efforts are also required to address chronic malnutrition in LMICs to avoid VAD-associated disabilities. In the current study, while significant reductions in VAD-associated mortalities were found across all regions, most Sub-Saharan African regions showed no significant improvements in VAD-associated morbidities and disabilities. While bi-annual high-dose vitamin A supplementation may serve as a critical intervention for children under 5 years of age, for children with a low dietary intake of vitamin A, high-dose vitamin A supplementation can only improve their vitamin A statuses for up to 3 months [7]. Thus, complementary vitamin A supplementation programs such as vitamin-A-fortified food production regimes are needed to maintain adequate vitamin A levels [21]. In Sub-Saharan African regions, the introduction of orange-fleshed sweet potatoes rich in vitamin A has been widely promoted [22]. However, coverage and household nutritional knowledge regarding the importance of consuming vitamin-A-rich foods have hindered the efforts to spread vitamin-A-fortified sweet potatoes across Sub-Saharan Africa [22]. Thus, policies that aim to improve household nutritional knowledge and expand the coverage of vitamin-A-fortified foods are required to effectively reduce the burden of vitamin A deficiency.

## 5. Conclusions

Significant improvements in VAD-associated mortalities have been identified across all regions and most nations, indicating the efficacy of wide-scale high-dose vitamin A supplementation for children under 5 years of age. However, the significant heterogeneity in national improvements highlights the need for more attention to be directed towards addressing nation-specific barriers that hinder optimal vitamin A supplementation in order to achieve universal vitamin A supplementation in LMICs. The need to continue crucial high-dose vitamin A supplementation has also been revealed, especially in low-SDI countries where VAD is endemic. Furthermore, Sub-Saharan Africa and countries with low SDIs showed no significant improvements in VAD morbidities. The lack of improvement in VAD morbidities in the last 30 years in vulnerable populations suggests the need to implement additional vitamin A supplementation programs to support children with suboptimal vitamin A intakes in order to reduce morbidity and mortality.

## Figures and Tables

**Table 1:** Vitamin A deficiency DALYs, mortality, and YLD across different SDI levels.

		1990	2021	AAPC	1990	2021	AAPC
		Rate	Rate		Number	Number	
DALYs (Disability-Adjusted Life Years)							
Global	<5 years	3035.82	310.85	−0.89 (−1.01, −0.75)	19,189,930.76	2,045,896.83	−0.89 (−1.02, −0.73)
Global	5–9 years	89.78	41.51	−0.53 (−0.61, −0.41)	525,363.08	285,213.87	−0.45 (−0.55, −0.31)
Global	10–14 years	55.5	27.54	−0.51 (−0.6, −0.41)	297,887.34	183,619.2	−0.39 (−0.51, −0.26)
Global	15–19 years	4.55	3.94	0.11 (0, 0.22)	23,647.31	24,613.78	0.34 (0.21, 0.46)
Global	<20 years	881.29	96.34	−0.88 (−1.08, −0.6)	2,003,6828.5	2,539,343.67	−0.86 (−1.09, −0.53)
Global	Age-standardized	783.77	98.38	-	-	-	-
High SDI	<5 years	22.66	1.42	−0.88 (−1.26, −0.42)	13,060.2	767.16	−0.89 (−1.23, −0.49)
High SDI	5–9 years	3.32	0.81	−0.82 (−0.89, −0.72)	1921.13	475.09	−0.83 (−0.89, −0.74)
High SDI	10–14 years	1.43	0.49	−0.8 (−0.88, −0.69)	819.46	294.22	−0.81 (−0.88, −0.7)
High SDI	15–19 years	0.04	0.08	0.18 (−0.1, 0.55)	22.08	47.51	0.08 (−0.18, 0.42)
High SDI	<20 years	6.76	0.68	−0.85 (−0.91, −0.6)	15,822.87	1583.98	−0.86 (−0.92, −0.63)
High SDI	Age-standardized	4.81	0.70	-	-	-	-
High-middle SDI	<5 years	218.74	12.43	−0.93 (−1.35, −0.46)	228,598.89	8704.19	−0.95 (−1.27, −0.59)
High-middle SDI	5–9 years	25.99	6.13	−0.73 (−0.8, −0.63)	26,205.71	5049.85	−0.75 (−0.82, −0.66)
High-middle SDI	10–14 years	15.37	3.9	−0.71 (−0.79, −0.59)	15,097.94	3055.52	−0.75 (−0.81, −0.65)
High-middle SDI	15–19 years	1.69	1.39	0.22 (0.01, 0.47)	1715.03	1009.01	−0.08 (−0.24, 0.1)
High-middle SDI	<20 years	67.02	5.87	−0.9 (−1.82, −0.38)	271,617.57	17,818.57	−0.92 (−1.68, −0.49)
High-middle SDI	Age-standardized	58.71	6.02	-	-	-	-
Middle SDI	<5 years	927.58	51.41	−0.94 (−1.25, −0.83)	1,899,988.42	90,794.97	−0.95 (−1.22, −0.85)
Middle SDI	5–9 years	59.39	17.06	−0.68 (−0.76, −0.57)	115,182.08	33,628.33	−0.68 (−0.76, −0.56)
Middle SDI	10–14 years	33.95	10.67	−0.67 (−0.75, −0.56)	619,95.21	20,615.7	−0.65 (−0.74, −0.54)
Middle SDI	15–19 years	5.06	3.91	0.08 (−0.07, 0.23)	9392.97	7121.89	0.05 (−0.09, 0.2)
Middle SDI	<20 years	272.07	20.31	−0.92 (−1.28, −0.43)	2,086,558.69	152,160.89	−0.92 (−1.27, −0.44)
Middle SDI	Age-standardized	253.55	21.16	-	-	-	-
Low-middle SDI	<5 years	4372.66	202.27	−0.95 (−1.06, −0.84)	7,355,065.09	387,507.39	−0.95 (−1.06, −0.83)
Low-middle SDI	5–9 years	136.66	47.23	−0.65 (−0.75, −0.49)	208,364.13	92,028.84	−0.58 (−0.7, −0.38)
Low-middle SDI	10–14 years	90.83	30.2	−0.67 (−0.77, −0.54)	120,548.99	58,406.99	−0.55 (−0.67, −0.36)
Low-middle SDI	15–19 years	6.69	4.89	−0.15 (−0.26, −0.03)	7835.06	9024.91	0.32 (0.15, 0.5)
Low-middle SDI	<20 years	1348.04	71.56	−0.94 (−1.12, −0.72)	7,691,813.28	546,968.14	−0.93 (−1.16, −0.64)
Low-middle SDI	Age-standardized	1124.76	72.63	-	-	-	-
Low SDI	<5 years	10028.33	940.12	−0.9 (−0.96, −0.81)	9,684,889.81	1,556,610.34	−0.82 (−0.92, −0.65)
Low SDI	5–9 years	217.54	100.22	−0.55 (−0.63, −0.45)	173,434.59	153,837.9	−0.09 (−0.26, 0.11)
Low SDI	10–14 years	151.09	71.65	−0.56 (−0.65, −0.45)	99,291.35	101,133.55	−0.01 (−0.2, 0.24)
Low SDI	15–19 years	8.75	5.96	−0.18 (−0.27, −0.05)	4670.96	7395.1	1.02 (0.78, 1.32)
Low SDI	<20 years	3372.81	311.36	−0.9 (−1.01, −0.76)	9,962,286.7	1,818,976.89	−0.8 (−1.02, −0.5)
Low SDI	Age-standardized	2593.93	285.92	-	-	-	-
Deaths							
Global	<5 years	32.83	2.64	−0.91 (−0.95, −0.86)	207,555.22	17,374.4	−0.91 (−0.95, −0.85)
Global	5–9 years	-	-	-	-	-	-
Global	10–14 years	-	-	-	-	-	-
Global	15–19 years	-	-	-	-	-	-
Global	<20 years	9.13	0.66	−0.92 (−0.96, −0.87)	207,555.22	17,374.4	−0.91 (−0.95, −0.85)
Global	Age-standardized	7.87	0.68	-	-	-	-
High SDI	<5 years	0.2	0	−0.96 (−0.99, −0.87)	115.25	1.14	−0.97 (−0.99, −0.89)
High SDI	5–9 years	-	-	-	-	-	-
High SDI	10–14 years	-	-	-	-	-	-
High SDI	15–19 years	-	-	-	-	-	-
High SDI	<20 years	0.05	0	−0.96 (−0.99, −0.88)	115.25	1.14	−0.97 (−0.99, −0.89)
High SDI	Age-standardized	0.01	0.001	-	-	-	-
High-middle SDI	<5 years	1.92	0.02	−0.99 (−1, −0.98)	2009.88	14.95	−0.99 (−1, −0.98)
High-middle SDI	5–9 years	-	-	-	-	-	-
High-middle SDI	10–14 years	-	-	-	-	-	-
High-middle SDI	15–19 years	-	-	-	-	-	-
High-middle SDI	<20 years	0.5	0	−0.99 (−1, −0.98)	2009.88	14.95	−0.99 (−1, −0.98)
High-middle SDI	Age-standardized	0.43	0.001	-	-	-	-
Middle SDI	<5 years	9.34	0.26	−0.97 (−0.99, −0.96)	19,134.99	454.33	−0.97 (−0.99, −0.96)
Middle SDI	5–9 years	-	-	-	-	-	-
Middle SDI	10–14 years	-	-	-	-	-	-
Middle SDI	15–19 years	-	-	-	-	-	-
Middle SDI	<20 years	2.5	0.06	−0.97 (−0.99, −0.96)	19,134.99	454.33	−0.97 (−0.99, −0.96)
Middle SDI	Age-standardized	2.27	0.07	-	-	-	-
Low-middle SDI	<5 years	47	1.36	−0.97 (−0.99, −0.95)	79,051.76	2604.91	−0.97 (−0.99, −0.94)
Low-middle SDI	5–9 years	-	-	-	-	-	-
Low-middle SDI	10–14 years	-	-	-	-	-	-
Low-middle SDI	15–19 years	-	-	-	-	-	-
Low-middle SDI	<20 years	13.85	0.34	−0.97 (−0.99, −0.95)	79,051.76	2604.91	−0.97 (−0.99, −0.94)
Low-middle SDI	Age-standardized	11.14	0.35	-	-	-	-
Low SDI	<5 years	110.95	8.63	−0.92 (−0.97, −0.85)	107,153.79	14,285.59	−0.85 (−0.94, −0.73)
Low SDI	5–9 years	-	-	-	-	-	-
Low SDI	10–14 years	-	-	-	-	-	-
Low SDI	15–19 years	-	-	-	-	-	-
Low SDI	<20 years	36.28	2.45	−0.93 (−0.97, −0.87)	107,153.79	14,285.59	−0.85 (−0.94, −0.73)
Low SDI	Age-standardized	26.96	2.23	-	-	-	-
YLD (Years Lived with Disability)							
Global	<5 years	182.48	77.24	−0.6 (−0.68, −0.39)	1,153,473.83	508,371.76	−0.58 (−0.66, −0.35)
Global	5–9 years	89.78	41.51	−0.53 (−0.61, −0.41)	525,363.08	285,213.87	−0.45 (−0.55, −0.31)
Global	10–14 years	55.5	27.54	−0.51 (−0.6, −0.41)	297,887.34	183,619.2	−0.39 (−0.51, −0.26)
Global	15–19 years	4.55	3.94	0.11 (0, 0.22)	23,647.31	24,613.78	0.34 (0.21, 0.46)
Global	<20 years	87.98	38.01	−0.58 (−0.65, −0.47)	2,000,371.57	1,001,818.6	−0.51 (−0.59, −0.38)
Global	Age-standardized	88.01	37.91	-	-	-	-
High SDI	<5 years	5.27	1.24	−0.82 (−0.86, −0.77)	3037.75	665.1	−0.84 (−0.88, −0.8)
High SDI	5–9 years	3.32	0.81	−0.82 (−0.89, −0.72)	1921.13	475.09	−0.83 (−0.89, −0.74)
High SDI	10–14 years	1.43	0.49	−0.8 (−0.88, −0.69)	819.46	294.22	−0.81 (−0.88, −0.7)
High SDI	15–19 years	0.04	0.08	0.18 (−0.1, 0.55)	22.08	47.51	0.08 (−0.18, 0.42)
High SDI	<20 years	2.48	0.64	−0.81 (−0.86, −0.77)	5800.42	1481.91	−0.83 (−0.87, −0.79)
High SDI	Age-standardized	3.54	0.65	-	-	-	-
High-middle SDI	<5 years	51.31	10.53	−0.76 (−0.82, −0.65)	53,621.55	7374.79	−0.82 (−0.86, −0.74)
High-middle SDI	5–9 years	25.99	6.13	−0.73 (−0.8, −0.63)	26,205.71	5049.85	−0.75 (−0.82, −0.66)
High-middle SDI	10–14 years	15.37	3.9	−0.71 (−0.79, −0.59)	15,097.94	3055.52	−0.75 (−0.81, −0.65)
High-middle SDI	15–19 years	1.69	1.39	0.22 (0.01, 0.47)	1715.03	1009.01	−0.08 (−0.24, 0.1)
High-middle SDI	<20 years	23.85	5.44	−0.73 (−0.78, −0.65)	96,640.24	16,489.18	−0.78 (−0.82, −0.72)
High-middle SDI	Age-standardized	20.56	5.53	-	-	-	-
Middle SDI	<5 years	114.27	28.53	−0.75 (−0.81, −0.6)	234,064.01	50,395.72	−0.78 (−0.83, −0.64)
Middle SDI	5–9 years	59.39	17.06	−0.68 (−0.76, −0.57)	115,182.08	33,628.33	−0.68 (−0.76, −0.56)
Middle SDI	10–14 years	33.95	10.67	−0.67 (−0.75, −0.56)	61,995.21	20,615.7	−0.65 (−0.74, −0.54)
Middle SDI	15–19 years	5.06	3.91	0.08 (−0.07, 0.23)	9392.97	7121.89	0.05 (−0.09, 0.2)
Middle SDI	<20 years	54.85	14.92	−0.71 (−0.77, −0.63)	420,634.28	111,761.64	−0.72 (−0.77, −0.64)
Middle SDI	Age-standardized	52.51	15.24	-	-	-	-
Low-middle SDI	<5 years	287.47	81.55	−0.74 (−0.79, −0.6)	483,542.87	156,227.62	−0.71 (−0.77, −0.56)
Low-middle SDI	5–9 years	136.66	47.23	−0.65 (−0.75, −0.49)	208,364.13	92,028.84	−0.58 (−0.7, −0.38)
Low-middle SDI	10–14 years	90.83	30.2	−0.67 (−0.77, −0.54)	120,548.99	58,406.99	−0.55 (−0.67, −0.36)
Low-middle SDI	15–19 years	6.69	4.89	−0.15 (−0.26, −0.03)	7835.06	9024.91	0.32 (0.15, 0.5)
Low-middle SDI	<20 years	143.76	41.3	−0.73 (−0.78, −0.65)	820,291.06	315,688.36	−0.64 (−0.71, −0.54)
Low-middle SDI	Age-standardized	139.48	41.38	-	-	-	-
Low SDI	<5 years	392.1	177.2	−0.59 (−0.67, −0.33)	378,676.48	293,391.14	−0.26 (−0.4, 0.22)
Low SDI	5–9 years	217.54	100.22	−0.55 (−0.63, −0.45)	173,434.59	153,837.9	−0.09 (−0.26, 0.11)
Low SDI	10–14 years	151.09	71.65	−0.56 (−0.65, −0.45)	99,291.35	101,133.55	−0.01 (−0.2, 0.24)
Low SDI	15–19 years	8.75	5.96	−0.18 (−0.27, −0.05)	4670.96	7395.1	1.02 (0.78, 1.32)
Low SDI	<20 years	222.12	95.13	−0.6 (−0.66, −0.49)	656,073.38	555,757.69	−0.17 (−0.3, 0.06)
Low SDI	Age-standardized	212.20	89.46	-	-	-	-

**Table 2:** Vitamin A deficiency DALYs, mortality, and YLD across different geographical regions.

		1990	2021	AAPC	1990	2021	AAPC
		Rate	Rate		Number	Number	
DALYs (Disability-Adjusted Life Years)							
Andean Latin America	<5 years	512.72	36.8	−0.94 (−1.46, −0.58)	27,925.53	2265.25	−0.93 (−1.54, −0.51)
Andean Latin America	5–9 years	60.89	20.1	−0.63 (−0.81, −0.11)	3028.29	1224.61	−0.54 (−0.76, 0.1)
Andean Latin America	10–14 years	33.73	10.69	−0.6 (−0.77, −0.35)	1559.76	624.99	−0.5 (−0.7, −0.17)
Andean Latin America	15–19 years	5.77	4.44	−0.07 (−0.43, 0.56)	235.99	247.59	0.26 (−0.23, 1.13)
Andean Latin America	<20 years	171.14	18.43	−0.9 (−1.51, 0.12)	32,749.57	4362.43	−0.87 (−1.64, 0.4)
Andean Latin America	Age-standardized	171.56	18.144	-	-	-	-
Australasia	<5 years	0.68	0.1	−0.74 (−0.89, −0.39)	10.52	1.8	−0.69 (−0.87, −0.28)
Australasia	5–9 years	0.36	0.05	−0.73 (−0.96, 1.19)	5.47	0.92	−0.65 (−0.95, 1.8)
Australasia	10–14 years	0.07	0.02	−0.67 (−0.88, −0.08)	1.12	0.36	−0.58 (−0.84, 0.19)
Australasia	15–19 years	0	0	-	0	0	-
Australasia	<20 years	0.27	0.04	−0.72 (−0.88, −0.31)	17.12	3.08	−0.67 (−0.85, −0.18)
Australasia	Age-standardized	0.16	0.04		-	-	-
Caribbean	<5 years	1052.15	366.3	−0.75 (−1.05, −0.63)	43,549.29	14,169.11	−0.77 (−1.04, −0.66)
Caribbean	5–9 years	52.06	55.14	−0.2 (−0.63,0.78)	1939.76	2125.92	−0.17 (−0.62, 0.84)
Caribbean	10–14 years	32.51	33.94	−0.23 (−0.62, 0.79)	1153.62	1283.47	−0.18 (−0.6, 0.9)
Caribbean	15–19 years	2.67	5.5	0.29 (−0.04, 0.73)	98.14	206.66	0.32 (−0.02, 0.77)
Caribbean	<20 years	309.78	116.53	−0.73 (−1.28, −0.42)	46,740.81	17,785.16	−0.73 (−1.28, −0.42)
Caribbean	Age-standardized	412.92	118.23	-	-	-	-
Central Asia	<5 years	310.86	61.11	−0.84 (−1.81, −0.32)	29,467.58	6109.18	−0.83 (−1.85, −0.29)
Central Asia	5–9 years	34.66	25.39	−0.55 (−0.78, −0.05)	2855.12	2394.32	−0.48 (−0.74, 0.09)
Central Asia	10–14 years	20.79	13.5	−0.51 (−0.71, −0.19)	1505.18	1114.04	−0.44 (−0.67, −0.08)
Central Asia	15–19 years	3.16	3.02	0.08 (−0.3, 0.77)	207.95	209.53	0.14 (−0.26, 0.86)
Central Asia	<20 years	107.91	28.38	−0.79 (−1.5, 0.25)	34,035.82	9827.06	−0.77 (−1.55, 0.37)
Central Asia	Age-standardized	118.34	26.23	-	-	-	-
Central Europe	<5 years	104.62	20.45	−0.85 (−1.62, −0.25)	9346.6	1142.44	−0.91 (−1.38, −0.54)
Central Europe	5–9 years	34.18	10.76	−0.78 (−0.85, −0.65)	3303.5	627.4	−0.87 (−0.91, −0.79)
Central Europe	10–14 years	18.49	7.29	−0.74 (−0.82, −0.62)	1912.88	458.14	−0.84 (−0.89, −0.77)
Central Europe	15–19 years	0	0	-	0	0	-
Central Europe	<20 years	37.77	9.46	−0.81 (−0.88, −0.29)	14,562.97	2227.97	−0.89 (−0.93, −0.57)
Central Europe	Age-standardized	53.43	9.81	-	-	-	-
Central Latin America	<5 years	537.8	39.35	−0.95 (−1.14, −0.82)	123,125.25	7904.76	−0.95 (−1.12, −0.84)
Central Latin America	5–9 years	45.31	11.01	−0.67 (−0.78, −0.53)	9568.59	2367.41	−0.66 (−0.77, −0.52)
Central Latin America	10–14 years	19.79	5.43	−0.6 (−0.72, −0.44)	3975.41	1188.47	−0.57 (−0.7, −0.39)
Central Latin America	15–19 years	5.43	2.1	−0.12 (−0.26, 0.03)	987.91	458.6	0.05 (−0.11, 0.23)
Central Latin America	<20 years	167.28	13.98	−0.94 (−1.3, −0.63)	137,657.16	11,919.24	−0.93 (−1.31, −0.62)
Central Latin America	Age-standardized	204.57	14.78	-	-	-	-
Central Sub-Saharan Africa	<5 years	8326.26	484.01	−0.92 (−1.03, −0.86)	895,069.5	101,964.93	−0.85 (−1.07, −0.72)
Central Sub-Saharan Africa	5–9 years	221.2	87.38	−0.45 (−0.66, −0.12)	18,371.54	17,407.03	0.33 (−0.16, 1.14)
Central Sub-Saharan Africa	10–14 years	158	87.63	−0.44 (−0.66, −0.05)	10,708.39	15,505.5	0.46 (−0.11, 1.48)
Central Sub-Saharan Africa	15–19 years	10.04	9.72	−0.17 (−0.4, 0.14)	570.68	1446.6	1.16 (0.56, 1.98)
Central Sub-Saharan Africa	<20 years	2934	185.32	−0.92 (−1.14, −0.75)	924,720.11	136,324.06	−0.8 (−1.34, −0.41)
Central Sub-Saharan Africa	Age-standardized	1730.88	170.06	-	-	-	-
East Asia	<5 years	366.4	11.91	−0.96 (−1.36, −0.72)	439,064.34	9540.66	−0.97 (−1.25, −0.81)
East Asia	5–9 years	28.47	6.57	−0.76 (−0.85, −0.62)	30,904.2	6461.42	−0.78 (−0.86, −0.65)
East Asia	10–14 years	20.65	4.54	−0.77 (−0.86, −0.61)	22,008.98	4034.04	−0.81 (−0.88, −0.68)
East Asia	15–19 years	2.59	2.59	0.17 (−0.09, 0.5)	3382.96	2009.07	−0.3 (−0.46, −0.1)
East Asia	<20 years	106.42	6.39	−0.93 (−1.41, −0.25)	495,360.48	22,045.18	−0.94 (−1.31, −0.44)
East Asia	Age-standardized	88.88	6.49	-	-	-	-
Eastern Europe	<5 years	3.28	0.59	−0.88 (−1.44, 0.41)	565.26	59.44	−0.93 (−1.26, −0.17)
Eastern Europe	5–9 years	1.06	0.28	−0.78 (−0.89, −0.58)	188.85	36.46	−0.84 (−0.92, −0.7)
Eastern Europe	10–14 years	0.54	0.23	−0.76 (−0.88, −0.54)	88.55	28.79	−0.82 (−0.91, −0.65)
Eastern Europe	15–19 years	0	0	-	0	0	-
Eastern Europe	<20 years	1.25	0.27	−0.85 (−0.93, −0.38)	842.65	124.69	−0.9 (−0.95, −0.57)
Eastern Europe	Age-standardized	1.80	0.28	-	-	-	-
Eastern Sub-Saharan Africa	<5 years	10505.72	978.72	−0.9 (−0.96, −0.81)	3,784,149.05	624,388.23	−0.82 (−0.93, −0.66)
Eastern Sub-Saharan Africa	5–9 years	210.32	96.66	−0.57 (−0.67, −0.45)	61,639.15	57,479.34	−0.13 (−0.32, 0.11)
Eastern Sub-Saharan Africa	10–14 years	146.42	60.8	−0.59 (−0.68, −0.48)	36,665.31	33,544.32	−0.1 (−0.3, 0.14)
Eastern Sub-Saharan Africa	15–19 years	8.46	6.13	−0.16 (−0.29, 0.01)	1714.69	3015.42	1.04 (0.73, 1.43)
Eastern Sub-Saharan Africa	<20 years	3510.89	315.67	−0.9 (−1, −0.76)	3,884,168.2	718,427.31	−0.8 (−1, −0.5)
Eastern Sub-Saharan Africa	Age-standardized	2582.32	293.25	-	-	-	-
High-income Asia Pacific	<5 years	6.12	0.77	−0.83 (−1.04, −0.63)	627.68	49.64	−0.89 (−1.03, −0.77)
High-income Asia Pacific	5–9 years	4.21	0.2	−0.84 (−0.93, −0.59)	501.23	15.36	−0.89 (−0.96, −0.73)
High-income Asia Pacific	10–14 years	2.42	0.15	−0.8 (−0.92, −0.51)	316.52	12.07	−0.88 (−0.95, −0.7)
High-income Asia Pacific	15–19 years	0	0	-	0	0	-
High-income Asia Pacific	<20 years	2.87	0.25	−0.82 (−0.91, −0.7)	1445.43	77.06	−0.89 (−0.94, −0.81)
High-income Asia Pacific	Age-standardized	1.66	0.29	-	-	-	-
High-income North America	<5 years	1.53	0.32	−0.74 (−1.29, 0.26)	328.98	66.17	−0.75 (−1.27, 0.19)
High-income North America	5–9 years	1.6	0.22	−0.7 (−0.86, −0.32)	328.18	49.17	−0.68 (−0.85, −0.27)
High-income North America	10–14 years	0.41	0.14	−0.49 (−0.79, 0.36)	78.65	31.56	−0.39 (−0.75, 0.62)
High-income North America	15–19 years	0	0	-	0	0	-
High-income North America	<20 years	0.9	0.16	−0.72 (−0.82, −0.37)	735.8	146.9	−0.69 (−0.8, −0.31)
High-income North America	Age-standardized	0.58	0.17	-	-	-	-
North Africa and Middle East	<5 years	2288.07	173.75	−0.9 (−1.07, −0.72)	1,220,495.35	106,226.65	−0.88 (−1.08, −0.67)
North Africa and Middle East	5–9 years	53.23	19.49	−0.6 (−0.71, −0.46)	25,554.22	12,318.68	−0.47 (−0.61, −0.28)
North Africa and Middle East	10–14 years	28.22	13.17	−0.59 (−0.68, −0.47)	11,958.69	7768.47	−0.42 (−0.55, −0.25)
North Africa and Middle East	15–19 years	3.02	4.35	0.11 (−0.14, 0.36)	1103.41	2312.9	0.62 (0.26, 0.99)
North Africa and Middle East	<20 years	698.36	54.39	−0.9 (−1.18, −0.65)	1,259,111.68	128,626.69	−0.86 (−1.24, −0.53)
North Africa and Middle East	Age-standardized	474.74	54.04	-	-	-	-
Oceania	<5 years	1785.76	327.31	−0.82 (−0.93, −0.68)	17,556.94	6331.61	−0.66 (−0.87, −0.38)
Oceania	5–9 years	79.24	43.21	−0.41 (−0.68, 0.13)	684.04	720.55	0.12 (−0.4, 1.14)
Oceania	10–14 years	46.49	23.56	−0.47 (−0.7, −0.02)	363.21	348.41	−0.01 (−0.43, 0.83)
Oceania	15–19 years	2.59	1.51	−0.23 (−0.46, 0.16)	17.71	19.71	0.47 (0.03, 1.2)
Oceania	<20 years	562.56	116.19	−0.8 (−1.04, −0.49)	18,621.91	7420.29	−0.62 (−1.07, −0.04)
Oceania	Age-standardized	506.95	101.69	-	-	-	-
South Asia	<5 years	3785.97	202.9	−0.95 (−1.07, −0.76)	6,124,618.99	321,792.25	−0.95 (−1.07, −0.76)
South Asia	5–9 years	136.39	52.35	−0.64 (−0.79, −0.36)	204,711.37	89,163.06	−0.58 (−0.76, −0.27)
South Asia	10–14 years	98.23	33.35	−0.69 (−0.81, −0.49)	124,864.8	59,403.94	−0.56 (−0.73, −0.28)
South Asia	15–19 years	8	5.13	−0.17 (−0.31, −0.04)	8723.81	9058.32	0.34 (0.12, 0.55)
South Asia	<20 years	1179.24	70.14	−0.94 (−1.16, −0.68)	6,462,918.98	479,417.57	−0.93 (−1.2, −0.59)
South Asia	Age-standardized	1138.23	74.89	-	-	-	-
Southeast Asia	<5 years	2725.84	102.77	−0.96 (−1.05, −0.88)	1,621,433.36	57,844.29	−0.96 (−1.05, −0.89)
Southeast Asia	5–9 years	113.02	22.08	−0.77 (−0.85, −0.65)	65,719.61	12,867.82	−0.77 (−0.85, −0.65)
Southeast Asia	10–14 years	54.36	12.9	−0.71 (−0.79, −0.6)	29,677.21	7492.37	−0.7 (−0.78, −0.57)
Southeast Asia	15–19 years	7.52	3.38	−0.09 (−0.21, 0.03)	3690.8	1911	0.05 (−0.09, 0.19)
Southeast Asia	<20 years	777.51	34.94	−0.95 (−1.08, −0.78)	1,720,520.98	80,115.48	−0.95 (−1.09, −0.77)
Southeast Asia	Age-standardized	746.24	36.18	-	-	-	-
Southern Latin America	<5 years	111.15	21.19	−0.82 (−2.01, 0.41)	5734.42	906.53	−0.85 (−1.84, 0.17)
Southern Latin America	5–9 years	22.85	6.29	−0.72 (−0.94, 0.64)	1140.45	324.23	−0.71 (−0.94, 0.7)
Southern Latin America	10–14 years	11.68	3.1	−0.69 (−0.89, −0.22)	558.27	156.84	−0.67 (−0.89, −0.18)
Southern Latin America	15–19 years	0	0	-	0	0	-
Southern Latin America	<20 years	38.37	7.11	−0.82 (−2.13, 0.03)	7433.14	1387.6	−0.82 (−2.14, 0.04)
Southern Latin America	Age-standardized	38.13	7.56	-	-	-	-
Southern Sub-Saharan Africa	<5 years	2206.68	390.7	−0.84 (−0.96, −0.79)	158,305.05	31,369.63	−0.83 (−0.95, −0.78)
Southern Sub-Saharan Africa	5–9 years	89.69	51.47	−0.53 (−0.73, −0.17)	6107.43	4197.96	−0.44 (−0.67, −0.02)
Southern Sub-Saharan Africa	10–14 years	49.43	29.29	−0.52 (−0.72, −0.14)	3159.54	2308.4	−0.41 (−0.65, 0.07)
Southern Sub-Saharan Africa	15–19 years	7.13	4	−0.14 (−0.37, 0.13)	416.02	287.63	0.07 (−0.22, 0.41)
Southern Sub-Saharan Africa	<20 years	640.98	122.07	−0.83 (−1.13, −0.54)	167,988.04	38,163.63	−0.8 (−1.15, −0.45)
Southern Sub-Saharan Africa	Age-standardized	667.92	121.80	-	-	-	-
Tropical Latin America	<5 years	903.04	45.73	−0.95 (−1.27, −0.82)	158,531.35	7868.89	−0.95 (−1.27, −0.82)
Tropical Latin America	5–9 years	80.15	31.5	−0.64 (−0.83, −0.25)	14,915.83	5262.44	−0.68 (−0.85, −0.33)
Tropical Latin America	10–14 years	50.19	20.08	−0.65 (−0.8, −0.35)	8923.55	3268.81	−0.68 (−0.82, −0.41)
Tropical Latin America	15–19 years	4.88	4.55	−0.13 (−0.28, 0.05)	764.98	745.43	−0.09 (−0.25, 0.1)
Tropical Latin America	<20 years	263	25.75	−0.91 (−1.72, −0.35)	183,135.7	17,145.55	−0.91 (−1.69, −0.37)
Tropical Latin America	Age-standardized	299.33	25.73	-	-	-	-
Western Europe	<5 years	5.44	1.52	−0.68 (−1.34, −0.45)	1248.37	321.91	−0.71 (−1.31, −0.49)
Western Europe	5–9 years	3.43	0.57	−0.76 (−0.85, −0.61)	807.8	130.84	−0.77 (−0.86, −0.62)
Western Europe	10–14 years	0.8	0.17	−0.8 (−0.87, −0.68)	195.57	41.51	−0.8 (−0.87, −0.69)
Western Europe	15–19 years	0	0	-	0	0	-
Western Europe	<20 years	2.29	0.54	−0.72 (−0.91, −0.61)	2251.73	494.25	−0.74 (−0.91, −0.64)
Western Europe	Age-standardized	1.99	0.49	-	-	-	-
Western Sub-Saharan Africa	<5 years	12695.24	932.46	−0.91 (−0.95, −0.85)	4,528,777.36	745,573.47	−0.8 (−0.88, −0.66)
Western Sub-Saharan Africa	5–9 years	255.81	97.89	−0.56 (−0.65, −0.45)	73,088.46	70,038.97	0.11 (−0.11, 0.37)
Western Sub-Saharan Africa	10–14 years	162.1	71.15	−0.53 (−0.63, −0.42)	38,212.13	45,004.7	0.25 (0, 0.56)
Western Sub-Saharan Africa	15–19 years	8.87	4.99	−0.26 (−0.37, −0.12)	1732.27	2685.31	1.03 (0.74, 1.41)
Western Sub-Saharan Africa	<20 years	4324.36	321.44	−0.91 (−0.97, −0.78)	4,641,810.22	863,302.46	−0.77 (−0.94, −0.44)
Western Sub-Saharan Africa	Age-standardized	2780.72	284.27	-	-	-	-
Deaths							
Andean Latin America	<5 years	4.8	0.13	−0.98 (−0.99, −0.97)	261.44	8.12	−0.97 (−0.98, −0.96)
Andean Latin America	<20 years	1.37	0.03	−0.98 (−0.99, −0.97)	261.44	8.12	−0.97 (−0.98, −0.96)
Andean Latin America	Age-standardized	1.40	0.03	-	-	-	-
Australasia	<5 years	0	0	−0.77 (−0.86, −0.64)	0	0	−0.73 (−0.84, −0.57)
Australasia	<20 years	0	0	−0.78 (−0.86, −0.64)	0	0	−0.73 (−0.84, −0.57)
Australasia	Age-standardized	0	0	-	-	-	-
Caribbean	<5 years	10.99	3.42	−0.78 (−0.91, −0.72)	454.74	132.11	−0.8 (−0.91, −0.74)
Caribbean	<20 years	3.01	0.87	−0.8 (−0.91, −0.74)	454.74	132.11	−0.8 (−0.91, −0.74)
Caribbean	Age-standardized	4.04	0.88	-	-	-	-
Central Asia	<5 years	2.69	0.34	−0.9 (−0.94, −0.86)	255.3	34.19	−0.89 (−0.94, −0.85)
Central Asia	<20 years	0.81	0.1	−0.9 (−0.94, −0.86)	255.3	34.19	−0.89 (−0.94, −0.85)
Central Asia	Age-standardized	0.87	0.09	-	-	-	-
Central Europe	<5 years	0.53	0.05	−0.93 (−1.01, −0.84)	47.71	2.82	−0.95 (−1, −0.9)
Central Europe	<20 years	0.12	0.01	−0.92 (−1.01, −0.84)	47.71	2.82	−0.95 (−1, −0.9)
Central Europe	Age-standardized	0.18	0.01	-	-	-	-
Central Latin America	<5 years	5.32	0.24	−0.97 (−0.98, −0.96)	1217.69	48.44	−0.97 (−0.99, −0.97)
Central Latin America	<20 years	1.48	0.06	−0.97 (−0.99, −0.97)	1217.69	48.44	−0.97 (−0.99, −0.97)
Central Latin America	Age-standardized	1.97	0.06	-	-	-	-
Central Sub-Saharan Africa	<5 years	91.4	3.36	−0.95 (−0.98, −0.86)	9825.86	707.38	−0.9 (−0.97, −0.73)
Central Sub-Saharan Africa	<20 years	31.18	0.96	−0.96 (−0.99, −0.88)	9825.86	707.38	−0.9 (−0.97, −0.73)
Central Sub-Saharan Africa	Age-standardized	17.59	0.87	-	-	-	-
East Asia	<5 years	3.57	0.01	−1 (−1, −1)	4274.36	7.95	−1 (−1, −1)
East Asia	<20 years	0.92	0	−1 (−1, - 1)	4274.36	7.95	−1 (−1, −1)
East Asia	Age-standardized	0.69	0.00	-	-	-	-
Eastern Europe	<5 years	0.01	0	−0.97 (−0.98, −0.96)	1.73	0.05	−0.98 (−0.99, −0.98)
Eastern Europe	<20 years	0	0	−0.98 (−0.98, −0.97)	1.73	0.05	−0.98 (−0.99, −0.98)
Eastern Europe	Age-standardized	0	0	-	-	-	-
Eastern Sub-Saharan Africa	<5 years	116.65	9.4	−0.91 (−0.97, −0.84)	42,015.64	5994.17	−0.84 (−0.94, −0.71)
Eastern Sub-Saharan Africa	<20 years	37.98	2.63	−0.92 (−0.97, −0.86)	42,015.64	5994.17	−0.84 (−0.94, −0.71)
Eastern Sub-Saharan Africa	Age-standardized	27.02	2.43	-	-	-	-
High-income Asia Pacific	<5 years	0.01	0	−0.94 (−0.96, −0.86)	0.97	0.06	−0.96 (−0.98, −0.91)
High-income Asia Pacific	<20 years	0	0	−0.93 (−0.96, −0.85)	0.97	0.06	−0.96 (−0.98, −0.91)
High-income Asia Pacific	Age-standardized	0	0	-	-	-	-
High-income North America	<5 years	0	0	−0.75 (−0.87, −0.53)	0.24	0.18	−0.76 (−0.88, −0.56)
High-income North America	<20 years	0	0	−0.78 (−0.89, −0.6)	0.24	0.18	−0.76 (−0.88, −0.56)
High-income North America	Age-standardized	0	0	-	-	-	-
North Africa and Middle East	<5 years	24.78	1.45	−0.92 (−0.97, −0.89)	13,219.21	885.63	−0.91 (−0.96, −0.86)
North Africa and Middle East	<20 years	7.33	0.37	−0.93 (−0.97, −0.9)	13,219.21	885.63	−0.91 (−0.96, −0.86)
North Africa and Middle East	Age-standardized	4.76	0.37	-	-	-	-
Oceania	<5 years	18.71	2.67	−0.86 (−1.01, −0.62)	183.92	51.68	−0.73 (−1.01, −0.27)
Oceania	<20 years	5.56	0.81	−0.86 (−1.01, −0.61)	183.92	51.68	−0.73 (−1.01, −0.27)
Oceania	Age-standardized	4.90	0.69	-	-	-	-
South Asia	<5 years	39.96	1.15	−0.97 (−1, −0.95)	64,647.38	1822.22	−0.97 (−1, −0.95)
South Asia	<20 years	11.8	0.27	−0.98 (−1, −0.96)	64,647.38	1822.22	−0.97 (−1, −0.95)
South Asia	Age-standardized	11.09	0.30	-	-	-	-
Southeast Asia	<5 years	28.83	0.66	−0.98 (−0.99, −0.95)	17,150.46	373.21	−0.98 (−0.99, −0.95)
Southeast Asia	<20 years	7.75	0.16	−0.98 (−0.99, −0.95)	17,150.46	373.21	−0.98 (−0.99, −0.95)
Southeast Asia	Age-standardized	7.34	0.17	-	-	-	-
Southern Latin America	<5 years	0.42	0.03	−0.95 (−0.97, −0.93)	21.91	1.27	−0.96 (−0.97, −0.94)
Southern Latin America	<20 years	0.11	0.01	−0.96 (−0.97, −0.94)	21.91	1.27	−0.96 (−0.97, −0.94)
Southern Latin America	Age-standardized	0.16	0.01	-	-	-	-
Southern Sub-Saharan Africa	<5 years	23.63	3.62	−0.86 (−0.95, −0.77)	1695.34	290.7	−0.85 (−0.94, −0.75)
Southern Sub-Saharan Africa	<20 years	6.47	0.93	−0.87 (−0.95, −0.79)	1695.34	290.7	−0.85 (−0.94, −0.75)
Southern Sub-Saharan Africa	Age-standardized	6.54	0.94	-	-	-	-
Tropical Latin America	<5 years	9.13	0.11	−0.99 (−0.99, −0.99)	1602.49	18.82	−0.99 (−0.99, −0.99)
Tropical Latin America	<20 years	2.3	0.03	−0.99 (−0.99, −0.98)	1602.49	18.82	−0.99 (−0.99, −0.99)
Tropical Latin America	Age-standardized	2.61	0.03	-	-	-	-
Western Europe	<5 years	0	0	−0.73 (−0.83, −0.65)	0.77	0.34	−0.75 (−0.85, −0.67)
Western Europe	<20 years	0	0	−0.74 (−0.84, −0.65)	0.77	0.34	−0.75 (−0.85, −0.67)
Western Europe	Age-standardized	0	0	-	-	-	-
Western Sub-Saharan Africa	<5 years	142.06	8.75	−0.92 (−0.98, −0.83)	50,678.07	6995.06	−0.83 (−0.96, −0.62)
Western Sub-Saharan Africa	<20 years	47.21	2.6	−0.93 (−0.98, −0.85)	50,678.07	6995.06	−0.83 (−0.96, −0.62)
Western Sub-Saharan Africa	Age-standardized	29.30	2.26	-	-	-	-
YLD (Years Lived with Disability)							
Andean Latin America	<5 years	93.69	25.08	−0.75 (−0.85, −0.35)	5102.94	1544.09	−0.71 (−0.83, −0.24)
Andean Latin America	5–9 years	60.89	20.1	−0.63 (−0.81, −0.11)	3028.29	1224.61	−0.54 (−0.76, 0.1)
Andean Latin America	10–14 years	33.73	10.69	−0.6 (−0.77, −0.35)	1559.76	624.99	−0.5 (−0.7, −0.17)
Andean Latin America	15–19 years	5.77	4.44	−0.07 (−0.43, 0.56)	235.99	247.59	0.26 (−0.23, 1.13)
Andean Latin America	<20 years	51.88	15.38	−0.69 (−0.79, −0.47)	9926.98	3641.27	−0.61 (−0.74, −0.34)
Andean Latin America	Age-standardized	47.74	15.11	-	-	-	-
Australasia	<5 years	0.67	0.09	−0.73 (−0.88, −0.37)	10.28	1.58	−0.68 (−0.86, −0.26)
Australasia	5–9 years	0.36	0.05	−0.73 (−0.96, 1.19)	5.47	0.92	−0.65 (−0.95, 1.8)
Australasia	10–14 years	0.07	0.02	−0.67 (−0.88, −0.08)	1.12	0.36	−0.58 (−0.84, 0.19)
Australasia	15–19 years	0	0	-	0	0	-
Australasia	<20 years	0.27	0.04	−0.72 (−0.88, −0.22)	16.87	2.86	−0.66 (−0.86, −0.06)
Australasia	Age-standardized	0.14	0.04	-	-	-	-
Caribbean	<5 years	89.43	62.19	−0.35 (−0.58, 0.21)	3701.68	2405.72	−0.39 (−0.61, 0.14)
Caribbean	5–9 years	52.06	55.14	−0.2 (−0.63, 0.78)	1939.76	2125.92	−0.17 (−0.62, 0.84)
Caribbean	10–14 years	32.51	33.94	−0.23 (−0.62, 0.79)	1153.62	1283.47	−0.18 (−0.6, 0.9)
Caribbean	15–19 years	2.67	5.5	0.29 (−0.04, 0.73)	98.14	206.66	0.32 (−0.02, 0.77)
Caribbean	<20 years	45.69	39.45	−0.27 (−0.51, 0.11)	6893.2	6021.77	−0.27 (−0.5, 0.12)
Caribbean	Age-standardized	53.52	39.52	-	-	-	-
Central Asia	<5 years	74.92	30.65	−0.58 (−0.71, −0.35)	7102.05	3064.21	−0.56 (−0.7, −0.32)
Central Asia	5–9 years	34.66	25.39	−0.55 (−0.78, −0.05)	2855.12	2394.32	−0.48 (−0.74, 0.09)
Central Asia	10–14 years	20.79	13.5	−0.51 (−0.71, −0.19)	1505.18	1114.04	−0.44 (−0.67, −0.08)
Central Asia	15–19 years	3.16	3.02	0.08 (−0.3, 0.77)	207.95	209.53	0.14 (−0.26, 0.86)
Central Asia	<20 years	37	19.59	−0.55 (−0.67, −0.35)	11,670.29	6782.09	−0.51 (−0.64, −0.28)
Central Asia	Age-standardized	40.58	18.35	-	-	-	-
Central Europe	<5 years	58.02	15.95	−0.78 (−0.82, −0.73)	5183.65	890.88	−0.87 (−0.89, −0.83)
Central Europe	5–9 years	34.18	10.76	−0.78 (−0.85, −0.65)	3303.5	627.4	−0.87 (−0.91, −0.79)
Central Europe	10–14 years	18.49	7.29	−0.74 (−0.82, −0.62)	1912.88	458.14	−0.84 (−0.89, −0.77)
Central Europe	15–19 years	0	0	-	0	0	-
Central Europe	<20 years	26.97	8.39	−0.77 (−0.81, −0.72)	10,400.03	1976.41	−0.86 (−0.89, −0.83)
Central Europe	Age-standardized	37.83	8.64	-	-	-	-
Central Latin America	<5 years	72.68	17.91	−0.72 (−0.8, −0.47)	16,639.5	3599.02	−0.76 (−0.83, −0.54)
Central Latin America	5–9 years	45.31	11.01	−0.67 (−0.78, −0.53)	9568.59	2367.41	−0.66 (−0.77, −0.52)
Central Latin America	10–14 years	19.79	5.43	−0.6 (−0.72, −0.44)	3975.41	1188.47	−0.57 (−0.7, −0.39)
Central Latin America	15–19 years	5.43	2.1	−0.12 (−0.26, 0.03)	987.91	458.6	0.05 (−0.11, 0.23)
Central Latin America	<20 years	37.88	8.93	−0.71 (−0.78, −0.59)	31,171.4	7613.5	−0.7 (−0.77, −0.57)
Central Latin America	Age-standardized	29.43	9.24	-	-	-	-
Central Sub-Saharan Africa	<5 years	375.64	185.96	−0.48 (−0.63, 0.15)	40,381.5	39,175.01	0.05 (−0.25, 1.33)
Central Sub-Saharan Africa	5–9 years	221.2	87.38	−0.45 (−0.66, −0.12)	18,371.54	17,407.03	0.33 (−0.16, 1.14)
Central Sub-Saharan Africa	10–14 years	158	87.63	−0.44 (−0.66, −0.05)	10,708.39	15,505.5	0.46 (−0.11, 1.48)
Central Sub-Saharan Africa	15–19 years	10.04	9.72	−0.17 (−0.4, 0.14)	570.68	1446.6	1.16 (0.56, 1.98)
Central Sub-Saharan Africa	<20 years	222.2	99.96	−0.5 (−0.62, −0.26)	70,032.12	73,534.15	0.19 (−0.09, 0.76)
Central Sub-Saharan Africa	Age-standardized	174.65	92.92	-	-	-	-
East Asia	<5 years	55.38	11.03	−0.8 (−0.88, −0.66)	66,367.9	8832.19	−0.86 (−0.91, −0.77)
East Asia	5–9 years	28.47	6.57	−0.76 (−0.85, −0.62)	30,904.2	6461.42	−0.78 (−0.86, −0.65)
East Asia	10–14 years	20.65	4.54	−0.77 (−0.86, −0.61)	22,008.98	4034.04	−0.81 (−0.88, −0.68)
East Asia	15–19 years	2.59	2.59	0.17 (−0.09, 0.5)	3382.96	2009.07	−0.3 (−0.46, −0.1)
East Asia	<20 years	26.35	6.19	−0.76 (−0.83, −0.67)	122,664.04	21,336.72	−0.82 (−0.87, −0.75)
East Asia	Age-standardized	27.29	6.26	-	-	-	-
Eastern Europe	<5 years	2.4	0.54	−0.83 (−0.89, −0.7)	413.29	54.67	−0.9 (−0.93, −0.82)
Eastern Europe	5–9 years	1.06	0.28	−0.78 (−0.89, −0.58)	188.85	36.46	−0.84 (−0.92, −0.7)
Eastern Europe	10–14 years	0.54	0.23	−0.76 (−0.88, −0.54)	88.55	28.79	−0.82 (−0.91, −0.65)
Eastern Europe	15–19 years	0	0	-	0	0	-
Eastern Europe	<20 years	1.03	0.26	−0.81 (−0.87, −0.72)	690.69	119.93	−0.87 (−0.91, −0.81)
Eastern Europe	Age-standardized	1.38	0.27	-	-	-	-
Eastern Sub-Saharan Africa	<5 years	367.94	148.43	−0.61 (−0.7, −0.26)	132,530.14	94,690.25	−0.32 (−0.47, 0.3)
Eastern Sub-Saharan Africa	5–9 years	210.32	96.66	−0.57 (−0.67, −0.45)	61,639.15	57,479.34	−0.13 (−0.32, 0.11)
Eastern Sub-Saharan Africa	10–14 years	146.42	60.8	−0.59 (−0.68, −0.48)	36,665.31	33,544.32	−0.1 (−0.3, 0.14)
Eastern Sub-Saharan Africa	15–19 years	8.46	6.13	−0.16 (−0.29, 0.01)	1714.69	3015.42	1.04 (0.73, 1.43)
Eastern Sub-Saharan Africa	<20 years	210.2	82.93	−0.62 (−0.69, −0.48)	232,549.3	188,729.33	−0.23 (−0.37, 0.06)
Eastern Sub-Saharan Africa	Age-standardized	195.80	78.35	-	-	-	-
High-income Asia Pacific	<5 years	5.3	0.69	−0.79 (−1.01, −0.6)	542.82	44.2	−0.87 (−1.01, −0.75)
High-income Asia Pacific	5–9 years	4.21	0.2	−0.84 (−0.93, −0.59)	501.23	15.36	−0.89 (−0.96, −0.73)
High-income Asia Pacific	10–14 years	2.42	0.15	−0.8 (−0.92, −0.51)	316.52	12.07	−0.88 (−0.95, −0.7)
High-income Asia Pacific	15–19 years	0	0	-	0	0	-
High-income Asia Pacific	<20 years	2.7	0.23	−0.8 (−0.91, −0.65)	1360.58	71.63	−0.88 (−0.95, −0.79)
High-income Asia Pacific	Age-standardized	1.33	0.26	-	-	-	-
High-income North America	<5 years	1.43	0.25	−0.73 (−0.86, −0.12)	307.9	50.24	−0.75 (−0.87, −0.17)
High-income North America	5–9 years	1.6	0.22	−0.7 (−0.86, −0.32)	328.18	49.17	−0.68 (−0.85, −0.27)
High-income North America	10–14 years	0.41	0.14	−0.49 (−0.79, 0.36)	78.65	31.56	−0.39 (−0.75, 0.62)
High-income North America	15–19 years	0	0	-	0	0	-
High-income North America	<20 years	0.88	0.15	−0.71 (−0.83, −0.45)	714.72	130.96	−0.68 (−0.81, −0.4)
High-income North America	Age-standardized	0.50	0.15	-	-	-	-
North Africa and Middle East	<5 years	131.52	45.6	−0.63 (−0.74, −0.28)	70,156.2	27,881.4	−0.56 (−0.69, −0.14)
North Africa and Middle East	5–9 years	53.23	19.49	−0.6 (−0.71, −0.46)	25,554.22	12,318.68	−0.47 (−0.61, −0.28)
North Africa and Middle East	10–14 years	28.22	13.17	−0.59 (−0.68, −0.47)	11,958.69	7768.47	−0.42 (−0.55, −0.25)
North Africa and Middle East	15–19 years	3.02	4.35	0.11 (−0.14, 0.36)	1103.41	2312.9	0.62 (0.26, 0.99)
North Africa and Middle East	<20 years	60.33	21.26	−0.63 (−0.72, −0.46)	108,772.53	50,281.44	−0.5 (−0.62, −0.27)
North Africa and Middle East	Age-standardized	53.23	20.87	-	-	-	-
Oceania	<5 years	160.46	90.25	−0.45 (−0.63, −0.07)	1577.61	1745.83	0.06 (−0.29, 0.79)
Oceania	5–9 years	79.24	43.21	−0.41 (−0.68, 0.13)	684.04	720.55	0.12 (−0.4, 1.14)
Oceania	10–14 years	46.49	23.56	−0.47 (−0.7, −0.02)	363.21	348.41	−0.01 (−0.43, 0.83)
Oceania	15–19 years	2.59	1.51	−0.23 (−0.46, 0.16)	17.71	19.71	0.47 (0.03, 1.2)
Oceania	<20 years	79.83	44.38	−0.44 (−0.59, −0.21)	2642.57	2834.5	0.07 (−0.23, 0.5)
Oceania	Age-standardized	72.44	40.34	-	-	-	-
South Asia	<5 years	316.52	100.92	−0.72 (−0.8, −0.58)	512,041.07	160,057.4	−0.71 (−0.8, −0.58)
South Asia	5–9 years	136.39	52.35	−0.64 (−0.79, −0.36)	204,711.37	89,163.06	−0.58 (−0.76, −0.27)
South Asia	10–14 years	98.23	33.35	−0.69 (−0.81, −0.49)	124,864.8	59,403.94	−0.56 (−0.73, −0.28)
South Asia	15–19 years	8	5.13	−0.17 (−0.31, −0.04)	8723.81	9058.32	0.34 (0.12, 0.55)
South Asia	<20 years	155.15	46.48	−0.72 (−0.79, −0.63)	850,341.05	317,682.72	−0.65 (−0.73, −0.54)
South Asia	Age-standardized	157.09	48.49	-	-	-	-
Southeast Asia	<5 years	215.77	43.84	−0.81 (−0.85, −0.67)	128,350.21	24,672.79	−0.82 (−0.85, −0.68)
Southeast Asia	5–9 years	113.02	22.08	−0.77 (−0.85, −0.65)	65,719.61	12,867.82	−0.77 (−0.85, −0.65)
Southeast Asia	10–14 years	54.36	12.9	−0.71 (−0.79, −0.6)	29,677.21	7492.37	−0.7 (−0.78, −0.57)
Southeast Asia	15–19 years	7.52	3.38	−0.09 (−0.21, 0.03)	3690.8	1911	0.05 (−0.09, 0.19)
Southeast Asia	<20 years	102.78	20.48	−0.79 (−0.83, −0.71)	227,437.83	46,943.98	−0.78 (−0.82, −0.7)
Southeast Asia	Age-standardized	96.35	20.92	-	-	-	-
Southern Latin America	<5 years	73.89	18.54	−0.7 (−0.88, −0.31)	3812.33	793.36	−0.75 (−0.9, −0.43)
Southern Latin America	5–9 years	22.85	6.29	−0.72 (−0.94, 0.64)	1140.45	324.23	−0.71 (−0.94, 0.7)
Southern Latin America	10–14 years	11.68	3.1	−0.69 (−0.89, −0.22)	558.27	156.84	−0.67 (−0.89, −0.18)
Southern Latin America	15–19 years	0	0	-	0	0	-
Southern Latin America	<20 years	28.45	6.53	−0.74 (−0.87, −0.4)	5511.05	1274.42	−0.73 (−0.87, −0.39)
Southern Latin America	Age-standardized	24.10	6.87	-	-	-	-
Southern Sub-Saharan Africa	<5 years	147.05	68.45	−0.59 (−0.72, 0.04)	10,549.4	5496.16	−0.56 (−0.7, 0.11)
Southern Sub-Saharan Africa	5–9 years	89.69	51.47	−0.53 (−0.73, −0.17)	6107.43	4197.96	−0.44 (−0.67, −0.02)
Southern Sub-Saharan Africa	10–14 years	49.43	29.29	−0.52 (−0.72, −0.14)	3159.54	2308.4	−0.41 (−0.65, 0.07)
Southern Sub-Saharan Africa	15–19 years	7.13	4	−0.14 (−0.37, 0.13)	416.02	287.63	0.07 (−0.22, 0.41)
Southern Sub-Saharan Africa	<20 years	77.2	39.31	−0.57 (−0.67, −0.32)	20,232.39	12,290.16	−0.49 (−0.61, −0.2)
Southern Sub-Saharan Africa	Age-standardized	86.96	38.40	-	-	-	-
Tropical Latin America	<5 years	99.53	35.98	−0.67 (−0.79, −0.24)	17,472.68	6190.79	−0.67 (−0.79, −0.23)
Tropical Latin America	5–9 years	80.15	31.5	−0.64 (−0.83, −0.25)	14,915.83	5262.44	−0.68 (−0.85, −0.33)
Tropical Latin America	10–14 years	50.19	20.08	−0.65 (−0.8, −0.35)	8923.55	3268.81	−0.68 (−0.82, −0.41)
Tropical Latin America	15–19 years	4.88	4.55	−0.13 (−0.28, 0.05)	764.98	745.43	−0.09 (−0.25, 0.1)
Tropical Latin America	<20 years	60.43	23.23	−0.65 (−0.75, −0.47)	42,077.03	15,467.46	−0.67 (−0.76, −0.49)
Tropical Latin America	Age-standardized	66.02	23.21	-	-	-	-
Western Europe	<5 years	5.14	1.37	−0.68 (−1.21, −0.54)	1181.18	291.62	−0.7 (−1.2, −0.58)
Western Europe	5–9 years	3.43	0.57	−0.76 (−0.85, −0.61)	807.8	130.84	−0.77 (−0.86, −0.62)
Western Europe	10–14 years	0.8	0.17	−0.8 (−0.87, −0.68)	195.57	41.51	−0.8 (−0.87, −0.69)
Western Europe	15–19 years	0	0	-	0	0	-
Western Europe	<20 years	2.22	0.51	−0.72 (−0.88, −0.59)	2184.54	463.96	−0.73 (−0.89, −0.62)
Western Europe	Age-standardized	1.85	0.45	-	-	-	-
Western Sub-Saharan Africa	<5 years	364.56	158.7	−0.58 (−0.67, −0.21)	130,049.51	126,890.35	−0.06 (−0.26, 0.78)
Western Sub-Saharan Africa	5–9 years	255.81	97.89	−0.56 (−0.65, −0.45)	73,088.46	70,038.97	0.11 (−0.11, 0.37)
Western Sub-Saharan Africa	10–14 years	162.1	71.15	−0.53 (−0.63, −0.42)	38,212.13	45,004.7	0.25 (0, 0.56)
Western Sub-Saharan Africa	15–19 years	8.87	4.99	−0.26 (−0.37, −0.12)	1732.27	2685.31	1.03 (0.74, 1.41)
Western Sub-Saharan Africa	<20 years	226.46	91.08	−0.58 (−0.65, −0.43)	243,082.37	244,619.34	0.04 (−0.12, 0.42)
Western Sub-Saharan Africa	Age-standardized	193.60	84	-	-	-	-

**Table 3:** National DALYs, mortality, and YLD for vitamin A deficiency.

		1990	2021
		ASR	ASR
DALYs (Disability-Adjusted Life Years)			
Afghanistan	Age-standardized	2669.53 (10,854.88, −7371.01)	347.22 (1319.32, −558.90)
Albania	Age-standardized	274.70 (802.25, −298.30)	24.06 (53.14, 5.77)
Algeria	Age-standardized	146.09 (513.26, −97.60)	15.40 (26.94, 7.63)
American Samoa	Age-standardized	60.88 (187.08, −22.47)	24.44 (72.44, −5.43)
Andorra	Age-standardized	1.57 (4.29, 0.06)	0.31 (0.81, 0.01)
Angola	Age-standardized	2979.88 (12,475.03, −9518.98)	132.17 (454.18, −124.37)
Antigua and Barbuda	Age-standardized	28.88 (60.53, 10.66)	13.72 (24.68, 7.06)
Argentina	Age-standardized	46.92 (137.69, −21.49)	9.65 (26.26, 2.48)
Armenia	Age-standardized	7.77 (33.19, −8.57)	0.75 (1.62, 0.22)
Australia	Age-standardized	0.03 (0.08, 0.01)	0.01 (0.01, 0.00)
Austria	Age-standardized	2.25 (6.23, −0.26)	0.37 (1.02, 0.01)
Azerbaijan	Age-standardized	50.29 (213.14, −71.96)	7.67 (23.87, −3.18)
Bahamas	Age-standardized	18.27 (36.77, 6.10)	11.33 (18.51, 5.88)
Bahrain	Age-standardized	13.76 (36.11, 0.13)	2.55 (5.52, 0.97)
Bangladesh	Age-standardized	616.94 (2512.48, −1221.03)	15.01 (35.67, 2.43)
Barbados	Age-standardized	17.75 (39.10, 5.89)	9.94 (18.32, 5.01)
Belarus	Age-standardized	3.35 (7.97, 0.63)	0.51 (1.18, 0.15)
Belgium	Age-standardized	2.22 (5.80, −0.06)	0.42 (1.12, −0.03)
Belize	Age-standardized	116.83 (413.61, −95.24)	23.21 (46.95, 8.69)
Benin	Age-standardized	3020.82 (11,879.69, −9604.18)	205.39 (737.17, −659.09)
Bermuda	Age-standardized	5.94 (13.74, 1.60)	1.23 (2.67, 0.42)
Bhutan	Age-standardized	549.74 (2425.32, −3646.54)	40.81 (107.77, −10.38)
Bolivia (Plurinational State of)	Age-standardized	240.41 (1064.34, −233.06)	30.65 (81.95, 3.54)
Bosnia and Herzegovina	Age-standardized	57.46 (114.96, 18.42)	15.02 (29.29, 6.01)
Botswana	Age-standardized	786.17 (3120.14, −1469.52)	123.25 (504.87, −155.63)
Brazil	Age-standardized	305.58 (1260.84, −610.85)	25.99 (50.89, 9.89)
Brunei Darussalam	Age-standardized	3.78 (7.97, 1.14)	0.79 (1.90, 0.15)
Bulgaria	Age-standardized	35.88 (74.66, 10.30)	13.81 (30.54, 4.02)
Burkina Faso	Age-standardized	3945.97 (15,424.43, −12,513.94)	415.23 (1472.99, −725.10)
Burundi	Age-standardized	1507.35 (5679.40, −4796.93)	122.17 (447.25, −402.34)
Cabo Verde	Age-standardized	1070.07 (4140.38, −1790.45)	30.14 (80.81, 1.83)
Cambodia	Age-standardized	743.40 (2861.37, −1728.40)	54.32 (130.09, 3.30)
Cameroon	Age-standardized	2179.27 (8279.14, −6331.14)	155.87 (520.38, −277.54)
Canada	Age-standardized	1.17 (3.39, −0.20)	0.23 (0.66, −0.03)
Central African Republic	Age-standardized	2663.91 (10,791.33, −8225.08)	530.80 (2127.93, −2093.95)
Chad	Age-standardized	4740.05 (20,103.75, −20,360.84)	1110.46 (5008.96, −5026.04)
Chile	Age-standardized	17.47 (58.48, −10.02)	1.97 (4.96, 0.04)
China	Age-standardized	90.81 (360.91, −155.06)	6.36 (10.19, 3.43)
Colombia	Age-standardized	58.10 (227.77, −65.03)	5.27 (11.40, 1.14)
Comoros	Age-standardized	1530.53 (5495.75, −3681.46)	115.99 (409.88, −101.51)
Congo	Age-standardized	1005.92 (4232.20, −4892.06)	188.32 (570.41, −427.00)
Cook Islands	Age-standardized	74.46 (199.56, −0.69)	10.05 (19.00, 4.36)
Costa Rica	Age-standardized	21.42 (65.36, −9.32)	4.53 (8.78, 1.76)
Croatia	Age-standardized	20.76 (40.28, 8.45)	6.45 (13.48, 1.77)
Cuba	Age-standardized	14.54 (38.18, −0.95)	3.95 (8.47, 1.46)
Cyprus	Age-standardized	6.45 (20.12, −1.02)	0.54 (1.60, −0.08)
Czechia	Age-standardized	22.46 (48.34, 5.60)	5.63 (14.00, 0.71)
Cote d’Ivoire	Age-standardized	1545.43 (5709.05, −4114.71)	154.45 (482.02, −214.91)
Democratic People’s Republic of Korea	Age-standardized	55.66 (105.95, 18.92)	17.07 (31.62, 7.18)
Democratic Republic of the Congo	Age-standardized	1394.31 (5294.99, −4185.64)	165.81 (528.45, −279.85)
Denmark	Age-standardized	1.51 (4.08, 0.03)	0.26 (0.73, −0.03)
Djibouti	Age-standardized	1200.68 (5028.34, −2805.15)	114.37 (401.61, −92.82)
Dominica	Age-standardized	28.22 (67.49, 4.26)	13.14 (23.19, 5.68)
Dominican Republic	Age-standardized	202.89 (861.48, −343.60)	24.91 (62.45, 3.69)
Ecuador	Age-standardized	137.15 (591.25, −202.74)	8.92 (16.84, 3.49)
Egypt	Age-standardized	248.24 (1123.53, −416.63)	12.97 (31.43, 1.51)
El Salvador	Age-standardized	297.28 (1351.48, −576.64)	12.59 (32.36, 1.46)
Equatorial Guinea	Age-standardized	2269.78 (9348.81, −10236.72)	47.82 (104.30, 1.28)
Eritrea	Age-standardized	2763.66 (10,990.59, −7762.82)	184.30 (707.12, −183.04)
Estonia	Age-standardized	2.50 (5.73, 0.50)	0.27 (0.57, 0.10)
Eswatini	Age-standardized	1197.12 (5042.29, −2713.19)	140.58 (604.67, −158.03)
Ethiopia	Age-standardized	3739.87 (14,156.19, −7822.82)	155.03 (519.03, −223.38)
Fiji	Age-standardized	138.18 (473.38, −88.55)	34.60 (93.80, 1.15)
Finland	Age-standardized	1.77 (4.28, 0.28)	0.34 (0.93, 0.02)
France	Age-standardized	0.47 (1.28, −0.01)	0.15 (0.42, −0.01)
Gabon	Age-standardized	370.88 (1405.33, −1029.48)	47.39 (109.25, −14.24)
Gambia	Age-standardized	2111.30 (7561.58, −5093.84)	242.02 (770.84, −158.19)
Georgia	Age-standardized	12.34 (43.09, −6.83)	3.80 (8.02, 1.63)
Germany	Age-standardized	0.56 (1.34, 0.06)	0.14 (0.36, 0.02)
Ghana	Age-standardized	1638.14 (5915.44, −4232.79)	134.80 (334.11, −15.27)
Greece	Age-standardized	3.05 (7.37, 0.50)	0.83 (2.06, 0.14)
Greenland	Age-standardized	3.31 (13.54, −1.63)	0.35 (0.83, 0.02)
Grenada	Age-standardized	56.22 (121.54, 9.32)	16.27 (28.49, 8.44)
Guam	Age-standardized	30.42 (84.05, −0.81)	8.43 (21.42, 0.83)
Guatemala	Age-standardized	515.80 (2222.44, −870.36)	31.60 (122.99, −26.70)
Guinea	Age-standardized	4248.75 (16,202.31, −11,064.29)	349.56 (1075.96, −607.75)
Guinea-Bissau	Age-standardized	4075.38 (15,124.00, −10,818.46)	481.82 (1615.26, −534.75)
Guyana	Age-standardized	162.98 (633.92, −197.59)	23.00 (50.79, 5.38)
Haiti	Age-standardized	1311.94 (6239.21, −3346.37)	262.91 (1024.86, −268.95)
Honduras	Age-standardized	258.37 (1163.50, −409.66)	18.43 (59.47, −1.47)
Hungary	Age-standardized	29.89 (62.52, 8.82)	9.32 (23.26, 0.72)
Iceland	Age-standardized	1.44 (3.75, 0.08)	0.28 (0.74, 0.01)
India	Age-standardized	1242.12 (4642.63, −2604.28)	91.75 (236.83, 3.65)
Indonesia	Age-standardized	1002.26 (4372.33, −3065.65)	46.75 (158.93, −30.20)
Iran (Islamic Republic of)	Age-standardized	51.35 (180.59, −104.83)	2.93 (5.70, 1.27)
Iraq	Age-standardized	172.95 (649.72, −207.37)	17.48 (34.28, 5.71)
Ireland	Age-standardized	2.07 (5.09, 0.42)	0.26 (0.70, 0.02)
Israel	Age-standardized	15.16 (35.59, 1.21)	3.72 (9.31, 0.38)
Italy	Age-standardized	2.74 (6.06, 0.85)	0.67 (1.57, 0.16)
Jamaica	Age-standardized	50.65 (147.02, −16.25)	16.20 (28.58, 8.32)
Japan	Age-standardized	1.14 (3.34, −0.06)	0.34 (1.03, −0.03)
Jordan	Age-standardized	51.05 (117.93, 5.25)	17.57 (30.83, 8.92)
Kazakhstan	Age-standardized	168.79 (605.16, −156.11)	27.65 (59.43, 11.07)
Kenya	Age-standardized	1325.62 (5352.39, −3979.83)	200.53 (708.38, −345.84)
Kiribati	Age-standardized	928.98 (3866.97, −2122.29)	179.23 (610.68, −186.74)
Kuwait	Age-standardized	14.10 (25.21, 6.48)	9.54 (16.08, 4.83)
Kyrgyzstan	Age-standardized	64.39 (252.60, −55.70)	18.64 (37.11, 7.44)
Lao People’s Democratic Republic	Age-standardized	2390.76 (10,169.18, −6028.17)	79.09 (281.60, −44.97)
Latvia	Age-standardized	2.35 (5.38, 0.51)	0.36 (0.77, 0.11)
Lebanon	Age-standardized	47.48 (182.61, −28.08)	3.75 (9.71, 0.15)
Lesotho	Age-standardized	1297.43 (6006.52, −3705.75)	377.68 (1820.88, −745.49)
Liberia	Age-standardized	3271.83 (13,033.20, −10,058.34)	207.29 (816.54, −480.44)
Libya	Age-standardized	74.49 (252.82, −62.49)	14.53 (24.76, 7.42)
Lithuania	Age-standardized	2.60 (6.26, 0.35)	0.28 (0.56, 0.11)
Luxembourg	Age-standardized	1.54 (3.80, 0.17)	0.24 (0.64, 0.01)
Madagascar	Age-standardized	2028.62 (8488.15, −5435.61)	232.41 (1145.09, −774.34)
Malawi	Age-standardized	2948.56 (11,448.21, −10,070.48)	180.83 (558.60, −215.23)
Malaysia	Age-standardized	46.18 (162.99, −24.06)	1.51 (3.50, 0.34)
Maldives	Age-standardized	641.34 (2574.75, −1753.70)	17.73 (40.32, 3.93)
Mali	Age-standardized	4782.93 (18,430.61, −16,555.06)	638.66 (1980.44, −934.96)
Malta	Age-standardized	3.95 (8.91, 0.83)	0.72 (1.86, 0.03)
Marshall Islands	Age-standardized	424.57 (1351.60, −495.10)	91.44 (193.14, 5.90)
Mauritania	Age-standardized	1280.17 (5071.43, −3340.42)	83.09 (253.04, −71.12)
Mauritius	Age-standardized	69.10 (180.26, −5.06)	10.38 (21.57, 3.74)
Mexico	Age-standardized	241.69 (1041.54, −440.66)	15.33 (37.74, 0.01)
Micronesia (Federated States of)	Age-standardized	417.63 (1265.60, −484.06)	129.00 (310.41, −7.29)
Monaco	Age-standardized	0.51 (1.44, 0.00)	0.13 (0.36, −0.01)
Mongolia	Age-standardized	48.57 (154.95, −30.09)	10.33 (18.54, 4.21)
Montenegro	Age-standardized	19.58 (38.72, 8.69)	7.24 (14.94, 3.11)
Morocco	Age-standardized	618.68 (2445.62, −1094.41)	28.70 (67.35, 1.20)
Mozambique	Age-standardized	3273.70 (12,526.25, −8941.39)	181.36 (629.03, −292.99)
Myanmar	Age-standardized	1991.05 (8157.65, −4992.28)	54.81 (161.95, −14.38)
Namibia	Age-standardized	591.67 (2362.44, −1069.78)	83.09 (324.75, −107.92)
Nauru	Age-standardized	133.26 (418.07, −74.22)	48.81 (126.12, 1.12)
Nepal	Age-standardized	1358.52 (5348.97, −2372.95)	32.64 (89.19, 5.27)
Netherlands	Age-standardized	1.22 (3.19, 0.08)	0.30 (0.92, −0.08)
New Zealand	Age-standardized	0.75 (2.13, 0.18)	0.21 (0.66, 0.05)
Nicaragua	Age-standardized	205.33 (979.42, −424.07)	5.38 (13.31, 0.50)
Niger	Age-standardized	6224.29 (25,142.50, −24,445.85)	679.22 (2578.14, −2718.01)
Nigeria	Age-standardized	2332.26 (9345.87, −5898.50)	168.24 (632.73, −377.88)
Niue	Age-standardized	80.28 (243.67, −24.54)	29.34 (84.31, −2.88)
North Macedonia	Age-standardized	147.00 (569.50, −255.39)	19.65 (44.03, 1.10)
Northern Mariana Islands	Age-standardized	22.63 (57.76, 1.02)	10.84 (25.17, 2.44)
Norway	Age-standardized	1.21 (2.68, 0.39)	0.14 (0.33, 0.04)
Oman	Age-standardized	89.08 (227.63, 17.28)	18.57 (30.51, 9.87)
Pakistan	Age-standardized	977.05 (3757.73, −1672.61)	43.59 (132.74, −6.02)
Palau	Age-standardized	63.35 (207.64, −55.41)	20.69 (54.26, −1.19)
Palestine	Age-standardized	206.49 (741.20, −236.24)	17.21 (29.07, 8.82)
Panama	Age-standardized	37.17 (146.42, −31.91)	7.94 (21.80, −0.92)
Papua New Guinea	Age-standardized	573.12 (2200.20, −1131.02)	101.76 (410.86, −161.41)
Paraguay	Age-standardized	129.83 (483.38, −125.40)	19.41 (41.31, 5.76)
Peru	Age-standardized	163.73 (645.55, −215.83)	18.41 (38.76, 6.82)
Philippines	Age-standardized	457.17 (1817.57, −929.92)	37.32 (115.55, −29.50)
Poland	Age-standardized	36.90 (69.45, 16.19)	7.17 (14.55, 2.70)
Portugal	Age-standardized	7.45 (19.39, −0.81)	1.11 (2.65, 0.22)
Puerto Rico	Age-standardized	7.92 (18.48, 2.41)	1.44 (3.41, 0.40)
Qatar	Age-standardized	12.47 (42.54, −3.51)	0.85 (1.88, 0.25)
Republic of Korea	Age-standardized	2.65 (6.79, 0.23)	0.15 (0.34, 0.03)
Republic of Moldova	Age-standardized	7.51 (24.23, −2.53)	1.14 (2.61, 0.28)
Romania	Age-standardized	53.34 (140.36, −11.66)	11.46 (24.37, 3.23)
Russian Federation	Age-standardized	0.92 (2.76, −0.21)	0.11 (0.23, 0.04)
Rwanda	Age-standardized	1469.12 (5429.29, −3271.21)	91.56 (335.15, −126.10)
Saint Kitts and Nevis	Age-standardized	47.85 (155.20, −16.25)	13.60 (25.32, 6.29)
Saint Lucia	Age-standardized	46.75 (123.55, −3.26)	15.90 (29.23, 7.86)
Saint Vincent and the Grenadines	Age-standardized	74.50 (242.56, −36.38)	19.79 (37.43, 9.00)
Samoa	Age-standardized	198.52 (661.68, −94.20)	42.79 (88.64, 17.12)
San Marino	Age-standardized	0.92 (2.43, 0.10)	0.23 (0.59, 0.02)
Sao Tome and Principe	Age-standardized	1982.48 (7390.01, −4355.70)	76.51 (188.20, 4.97)
Saudi Arabia	Age-standardized	29.95 (121.71, −28.56)	0.51 (1.14, 0.15)
Senegal	Age-standardized	2006.62 (7466.52, −6100.96)	76.92 (230.49, −14.37)
Serbia	Age-standardized	76.24 (160.42, 22.57)	17.14 (33.91, 6.75)
Seychelles	Age-standardized	50.62 (159.96, −12.82)	7.26 (14.66, 2.79)
Sierra Leone	Age-standardized	3401.02 (13,721.12, −12,982.25)	216.49 (818.86, −502.22)
Singapore	Age-standardized	2.36 (6.55, 0.26)	0.22 (0.52, 0.08)
Slovakia	Age-standardized	28.13 (52.96, 12.09)	6.92 (14.20, 2.30)
Slovenia	Age-standardized	14.08 (28.09, 6.35)	3.61 (7.12, 1.39)
Solomon Islands	Age-standardized	768.77 (2888.77, −1367.89)	168.30 (483.72, −51.21)
Somalia	Age-standardized	5437.19 (21,484.44, −16,752.38)	2199.05 (9108.03, −5903.07)
South Africa	Age-standardized	671.07 (2866.16, −1158.85)	81.70 (310.31, −70.41)
South Sudan	Age-standardized	3464.03 (13,992.17, −8056.73)	708.43 (3175.29, −1759.12)
Spain	Age-standardized	1.96 (4.78, 0.27)	0.40 (1.11, −0.01)
Sri Lanka	Age-standardized	111.88 (345.39, −108.77)	10.35 (19.65, 4.15)
Sudan	Age-standardized	1678.67 (6916.70, −2998.04)	62.84 (207.06, 4.04)
Suriname	Age-standardized	104.31 (371.90, −124.89)	29.40 (69.01, 5.45)
Sweden	Age-standardized	1.18 (2.74, 0.36)	0.23 (0.57, 0.04)
Switzerland	Age-standardized	0.73 (2.15, −0.03)	0.13 (0.38, 0.00)
Syrian Arab Republic	Age-standardized	170.72 (612.69, −195.12)	11.42 (23.99, 4.57)
Taiwan (Province of China)	Age-standardized	5.69 (14.02, 0.43)	0.45 (1.16, 0.06)
Tajikistan	Age-standardized	330.62 (1437.71, −650.24)	82.53 (303.79, −92.08)
Thailand	Age-standardized	201.15 (715.64, −144.52)	9.71 (18.57, 3.64)
Timor-Leste	Age-standardized	1393.89 (6619.08, −4530.19)	102.28 (298.95, −59.55)
Togo	Age-standardized	1845.41 (7463.01, −5905.06)	199.94 (652.97, −410.86)
Tokelau	Age-standardized	193.76 (677.43, −220.19)	76.48 (309.52, −89.91)
Tonga	Age-standardized	181.46 (590.16, −122.58)	29.41 (59.76, 11.01)
Trinidad and Tobago	Age-standardized	25.59 (61.65, 3.89)	11.46 (19.35, 5.90)
Tunisia	Age-standardized	59.96 (201.35, −20.91)	9.73 (16.46, 4.86)
Turkey	Age-standardized	211.10 (853.45, −290.09)	7.10 (14.94, 2.37)
Turkmenistan	Age-standardized	108.12 (454.65, −143.27)	14.66 (26.30, 6.62)
Tuvalu	Age-standardized	613.19 (2195.24, −890.23)	71.23 (190.06, −6.98)
Uganda	Age-standardized	1896.43 (7273.59, −3911.83)	63.82 (198.71, −91.96)
Ukraine	Age-standardized	3.44 (8.68, 0.29)	0.86 (1.77, 0.31)
United Arab Emirates	Age-standardized	13.61 (46.93, −2.06)	1.38 (3.19, 0.37)
United Kingdom	Age-standardized	2.16 (4.72, 0.71)	0.46 (1.09, 0.17)
United Republic of Tanzania	Age-standardized	1207.12 (4676.68, −2505.43)	112.92 (358.65, −126.20)
United States of America	Age-standardized	0.52 (1.30, 0.05)	0.17 (0.38, 0.05)
United States Virgin Islands	Age-standardized	10.10 (25.52, 2.08)	1.92 (4.46, 0.61)
Uruguay	Age-standardized	34.83 (112.42, −19.55)	6.40 (15.45, 0.42)
Uzbekistan	Age-standardized	94.88 (356.30, −81.56)	17.39 (33.21, 8.41)
Vanuatu	Age-standardized	573.16 (1993.44, −1016.81)	169.77 (520.53, −146.92)
Venezuela (Bolivarian Republic of)	Age-standardized	88.81 (375.18, −113.78)	19.20 (61.51, −3.08)
Vietnam	Age-standardized	228.01 (834.21, −206.64)	10.11 (18.30, 4.72)
Yemen	Age-standardized	2121.46 (8478.60, −4449.96)	116.92 (317.07, 11.86)
Zambia	Age-standardized	2284.07 (9076.55, −4730.44)	155.61 (492.86, −161.78)
Zimbabwe	Age-standardized	523.58 (1862.25, −755.73)	193.72 (689.76, −102.21)
Deaths			
Afghanistan	Age-standardized	28.67 (120.19, −84.02)	3.24 (14.00, −6.82)
Albania	Age-standardized	1.95 (7.06, −3.86)	0.04 (0.21, −0.07)
Algeria	Age-standardized	1.19 (5.03, −1.36)	0.01 (0.05, −0.01)
American Samoa	Age-standardized	0.39 (1.67, −0.44)	0.14 (0.59, −0.14)
Andorra	Age-standardized	0.00 (0.01, 0.00)	0.00 (0.00, 0.00)
Angola	Age-standardized	32.28 (138.62, −107.60)	0.88 (4.17, −1.82)
Antigua and Barbuda	Age-standardized	0.03 (0.16, −0.05)	0.00 (0.02, −0.01)
Argentina	Age-standardized	0.19 (0.92, −0.38)	0.01 (0.05, −0.02)
Armenia	Age-standardized	0.07 (0.33, −0.11)	0.00 (0.00, 0.00)
Australia	Age-standardized	0.00 (0.00, 0.00)	0.00 (0.00, 0.00)
Austria	Age-standardized	0.00 (0.01, 0.00)	0.00 (0.00, 0.00)
Azerbaijan	Age-standardized	0.43 (2.14, −0.88)	0.03 (0.17, −0.07)
Bahamas	Age-standardized	0.03 (0.13, −0.04)	0.00 (0.01, 0.00)
Bahrain	Age-standardized	0.04 (0.19, −0.07)	0.00 (0.01, 0.00)
Bangladesh	Age-standardized	5.92 (26.53, −14.54)	0.04 (0.20, −0.06)
Barbados	Age-standardized	0.02 (0.11, −0.03)	0.00 (0.01, 0.00)
Belarus	Age-standardized	0.01 (0.03, −0.01)	0.00 (0.00, 0.00)
Belgium	Age-standardized	0.00 (0.02, 0.00)	0.00 (0.00, 0.00)
Belize	Age-standardized	0.78 (3.78, −1.42)	0.03 (0.14, −0.04)
Benin	Age-standardized	31.77 (130.63, −109.83)	0.95 (6.41, −8.49)
Bermuda	Age-standardized	0.01 (0.04, −0.01)	0.00 (0.00, 0.00)
Bhutan	Age-standardized	4.41 (24.93, −42.54)	0.10 (0.58, −0.32)
Bolivia (Plurinational State of)	Age-standardized	2.15 (11.06, −2.97)	0.09 (0.47, −0.11)
Bosnia and Herzegovina	Age-standardized	0.07 (0.32, −0.13)	0.01 (0.07, −0.02)
Botswana	Age-standardized	7.37 (32.53, −17.42)	0.92 (4.82, −2.07)
Brazil	Age-standardized	2.67 (12.85, −7.22)	0.03 (0.15, −0.05)
Brunei Darussalam	Age-standardized	0.00 (0.02, −0.01)	0.00 (0.00, 0.00)
Bulgaria	Age-standardized	0.04 (0.21, −0.08)	0.02 (0.10, −0.03)
Burkina Faso	Age-standardized	40.75 (169.03, −143.48)	3.16 (14.07, −9.30)
Burundi	Age-standardized	15.23 (61.28, −55.28)	0.66 (3.85, −5.01)
Cabo Verde	Age-standardized	10.63 (44.19, −20.93)	0.09 (0.44, −0.12)
Cambodia	Age-standardized	6.16 (28.72, −20.67)	0.11 (0.62, −0.26)
Cameroon	Age-standardized	22.36 (89.96, −72.76)	1.01 (4.61, −3.66)
Canada	Age-standardized	0.00 (0.01, 0.00)	0.00 (0.00, 0.00)
Central African Republic	Age-standardized	27.38 (117.66, −93.96)	4.09 (21.14, −24.72)
Chad	Age-standardized	49.81 (221.88, −231.91)	10.41 (53.24, −58.40)
Chile	Age-standardized	0.09 (0.43, −0.16)	0.00 (0.02, −0.01)
China	Age-standardized	0.72 (3.60, −1.93)	0.00 (0.01, −0.01)
Colombia	Age-standardized	0.46 (2.19, −0.83)	0.01 (0.06, −0.02)
Comoros	Age-standardized	15.35 (58.32, −42.33)	0.72 (3.47, −1.52)
Congo	Age-standardized	9.05 (43.80, −55.81)	0.52 (3.86, −5.78)
Cook Islands	Age-standardized	0.35 (1.46, −0.35)	0.01 (0.03, −0.01)
Costa Rica	Age-standardized	0.10 (0.50, −0.17)	0.00 (0.02, −0.01)
Croatia	Age-standardized	0.02 (0.09, −0.04)	0.01 (0.05, −0.02)
Cuba	Age-standardized	0.05 (0.22, −0.08)	0.00 (0.01, 0.00)
Cyprus	Age-standardized	0.02 (0.13, −0.03)	0.00 (0.00, 0.00)
Czechia	Age-standardized	0.03 (0.17, −0.07)	0.01 (0.07, −0.02)
Cote d’Ivoire	Age-standardized	15.22 (61.19, −47.84)	0.85 (4.05, −3.01)
Democratic People’s Republic of Korea	Age-standardized	0.06 (0.31, −0.14)	0.01 (0.04, −0.01)
Democratic Republic of the Congo	Age-standardized	13.70 (56.46, −48.08)	0.70 (4.21, −3.93)
Denmark	Age-standardized	0.00 (0.01, 0.00)	0.00 (0.00, 0.00)
Djibouti	Age-standardized	12.07 (53.89, −32.26)	0.78 (3.67, −1.39)
Dominica	Age-standardized	0.06 (0.35, −0.12)	0.01 (0.06, −0.02)
Dominican Republic	Age-standardized	1.75 (8.81, −4.11)	0.08 (0.40, −0.08)
Ecuador	Age-standardized	1.22 (5.91, −2.36)	0.01 (0.06, −0.02)
Egypt	Age-standardized	2.44 (11.95, −4.82)	0.03 (0.17, −0.05)
El Salvador	Age-standardized	2.89 (14.31, −6.70)	0.04 (0.21, −0.05)
Equatorial Guinea	Age-standardized	22.58 (100.90, −116.71)	0.09 (0.48, −0.29)
Eritrea	Age-standardized	28.60 (119.82, −88.97)	1.18 (6.24, −2.63)
Estonia	Age-standardized	0.00 (0.02, −0.01)	0.00 (0.00, 0.00)
Eswatini	Age-standardized	12.23 (54.57, −31.03)	1.14 (6.01, −2.03)
Ethiopia	Age-standardized	39.86 (156.63, −89.92)	0.97 (4.75, −3.03)
Fiji	Age-standardized	0.99 (4.46, −1.30)	0.11 (0.49, −0.14)
Finland	Age-standardized	0.00 (0.00, 0.00)	0.00 (0.00, 0.00)
France	Age-standardized	0.00 (0.00, 0.00)	0.00 (0.00, 0.00)
Gabon	Age-standardized	2.99 (13.88, −12.43)	0.06 (0.37, −0.47)
Gambia	Age-standardized	20.89 (81.14, −59.18)	1.44 (6.49, −2.63)
Georgia	Age-standardized	0.07 (0.35, −0.11)	0.00 (0.00, 0.00)
Germany	Age-standardized	0.00 (0.00, 0.00)	0.00 (0.00, 0.00)
Ghana	Age-standardized	15.89 (62.88, −49.22)	0.40 (1.97, −0.99)
Greece	Age-standardized	0.00 (0.00, 0.00)	0.00 (0.00, 0.00)
Greenland	Age-standardized	0.03 (0.13, −0.02)	0.00 (0.00, 0.00)
Grenada	Age-standardized	0.12 (0.59, −0.24)	0.00 (0.03, −0.01)
Guam	Age-standardized	0.14 (0.64, −0.12)	0.02 (0.12, −0.03)
Guatemala	Age-standardized	5.35 (24.27, −10.01)	0.23 (1.20, −0.39)
Guinea	Age-standardized	45.32 (178.93, −126.66)	2.64 (10.20, −7.70)
Guinea-Bissau	Age-standardized	43.07 (166.44, −124.12)	4.14 (16.10, −7.02)
Guyana	Age-standardized	1.19 (6.07, −2.74)	0.05 (0.22, −0.08)
Haiti	Age-standardized	13.27 (67.38, −38.70)	2.04 (9.85, −3.60)
Honduras	Age-standardized	2.56 (12.49, −4.80)	0.10 (0.49, −0.09)
Hungary	Age-standardized	0.04 (0.21, −0.08)	0.03 (0.13, −0.04)
Iceland	Age-standardized	0.00 (0.00, 0.00)	0.00 (0.00, 0.00)
India	Age-standardized	12.11 (49.77, −30.48)	0.36 (1.68, −0.41)
Indonesia	Age-standardized	9.90 (46.99, −35.03)	0.27 (1.37, −0.47)
Iran (Islamic Republic of)	Age-standardized	0.26 (1.44, −1.34)	0.00 (0.01, 0.00)
Iraq	Age-standardized	1.43 (6.41, −2.65)	0.03 (0.14, −0.05)
Ireland	Age-standardized	0.00 (0.00, 0.00)	0.00 (0.00, 0.00)
Israel	Age-standardized	0.02 (0.09, −0.04)	0.00 (0.02, −0.01)
Italy	Age-standardized	0.00 (0.00, 0.00)	0.00 (0.00, 0.00)
Jamaica	Age-standardized	0.22 (1.12, −0.41)	0.01 (0.04, −0.01)
Japan	Age-standardized	0.00 (0.01, 0.00)	0.00 (0.00, 0.00)
Jordan	Age-standardized	0.10 (0.55, −0.20)	0.01 (0.05, −0.01)
Kazakhstan	Age-standardized	0.97 (4.90, −2.23)	0.01 (0.04, −0.02)
Kenya	Age-standardized	13.26 (57.60, −45.69)	1.48 (6.82, −4.41)
Kiribati	Age-standardized	8.91 (41.05, −24.96)	1.12 (5.42, −2.63)
Kuwait	Age-standardized	0.01 (0.04, −0.01)	0.00 (0.00, 0.00)
Kyrgyzstan	Age-standardized	0.44 (2.28, −0.78)	0.02 (0.10, −0.04)
Lao People’s Democratic Republic	Age-standardized	24.99 (111.50, −68.83)	0.44 (2.39, −0.80)
Latvia	Age-standardized	0.00 (0.02, 0.00)	0.00 (0.00, 0.00)
Lebanon	Age-standardized	0.37 (1.72, −0.40)	0.01 (0.06, −0.01)
Lesotho	Age-standardized	13.09 (64.98, −42.41)	3.58 (19.24, −8.75)
Liberia	Age-standardized	34.76 (143.61, −114.64)	1.53 (8.04, −5.96)
Libya	Age-standardized	0.53 (2.36, −0.87)	0.01 (0.05, −0.01)
Lithuania	Age-standardized	0.00 (0.03, −0.01)	0.00 (0.00, 0.00)
Luxembourg	Age-standardized	0.00 (0.01, 0.00)	0.00 (0.00, 0.00)
Madagascar	Age-standardized	21.16 (92.88, −62.27)	1.91 (11.68, −9.24)
Malawi	Age-standardized	30.47 (124.78, −115.11)	1.00 (4.49, −3.04)
Malaysia	Age-standardized	0.37 (1.62, −0.38)	0.00 (0.01, 0.00)
Maldives	Age-standardized	4.93 (25.09, −21.05)	0.03 (0.15, −0.05)
Mali	Age-standardized	50.25 (203.10, −190.00)	5.22 (19.07, −11.99)
Malta	Age-standardized	0.00 (0.00, 0.00)	0.00 (0.00, 0.00)
Marshall Islands	Age-standardized	3.16 (12.72, −6.47)	0.20 (0.98, −0.52)
Mauritania	Age-standardized	12.43 (54.20, −39.00)	0.38 (1.79, −1.18)
Mauritius	Age-standardized	0.19 (0.97, −0.37)	0.01 (0.05, −0.02)
Mexico	Age-standardized	2.32 (11.02, −5.16)	0.05 (0.24, −0.09)
Micronesia (Federated States of)	Age-standardized	2.82 (11.43, −6.67)	0.46 (1.97, −0.79)
Monaco	Age-standardized	0.00 (0.00, 0.00)	0.00 (0.00, 0.00)
Mongolia	Age-standardized	0.26 (1.29, −0.52)	0.01 (0.05, −0.02)
Montenegro	Age-standardized	0.01 (0.03, −0.01)	0.00 (0.00, 0.00)
Morocco	Age-standardized	6.12 (25.98, −12.69)	0.07 (0.37, −0.17)
Mozambique	Age-standardized	34.15 (137.43, −102.28)	0.96 (5.41, −4.11)
Myanmar	Age-standardized	20.41 (88.55, −57.29)	0.20 (1.04, −0.37)
Namibia	Age-standardized	5.65 (24.65, −12.66)	0.59 (3.05, −1.47)
Nauru	Age-standardized	0.81 (3.60, −1.33)	0.13 (0.66, −0.22)
Nepal	Age-standardized	13.93 (58.00, −27.44)	0.10 (0.48, −0.09)
Netherlands	Age-standardized	0.00 (0.00, 0.00)	0.00 (0.00, 0.00)
New Zealand	Age-standardized	0.00 (0.00, 0.00)	0.00 (0.00, 0.00)
Nicaragua	Age-standardized	2.02 (10.43, −4.86)	0.02 (0.09, −0.02)
Niger	Age-standardized	66.22 (277.69, −278.63)	4.90 (25.43, −32.64)
Nigeria	Age-standardized	25.29 (104.02, −67.48)	1.50 (6.54, −4.58)
Niue	Age-standardized	0.40 (1.92, −0.58)	0.14 (0.66, −0.15)
North Macedonia	Age-standardized	1.04 (5.25, −3.19)	0.04 (0.20, −0.09)
Northern Mariana Islands	Age-standardized	0.08 (0.37, −0.07)	0.02 (0.10, −0.03)
Norway	Age-standardized	0.00 (0.00, 0.00)	0.00 (0.00, 0.00)
Oman	Age-standardized	0.28 (1.52, −0.29)	0.00 (0.02, −0.01)
Pakistan	Age-standardized	9.54 (40.02, −19.68)	0.21 (1.09, −0.25)
Palau	Age-standardized	0.37 (1.74, −0.84)	0.07 (0.35, −0.11)
Palestine	Age-standardized	1.71 (7.22, −3.05)	0.01 (0.04, −0.01)
Panama	Age-standardized	0.26 (1.36, −0.43)	0.03 (0.15, −0.05)
Papua New Guinea	Age-standardized	5.74 (23.47, −13.16)	0.73 (3.86, −2.07)
Paraguay	Age-standardized	0.89 (4.32, −1.55)	0.03 (0.17, −0.05)
Peru	Age-standardized	1.21 (6.08, −2.65)	0.02 (0.13, −0.04)
Philippines	Age-standardized	4.48 (19.27, −10.68)	0.17 (0.91, −0.48)
Poland	Age-standardized	0.02 (0.09, −0.04)	0.01 (0.03, −0.01)
Portugal	Age-standardized	0.02 (0.07, −0.03)	0.00 (0.00, 0.00)
Puerto Rico	Age-standardized	0.01 (0.04, −0.01)	0.00 (0.01, 0.00)
Qatar	Age-standardized	0.08 (0.37, −0.08)	0.00 (0.00, 0.00)
Republic of Korea	Age-standardized	0.01 (0.04, −0.01)	0.00 (0.00, 0.00)
Republic of Moldova	Age-standardized	0.03 (0.16, −0.05)	0.00 (0.00, 0.00)
Romania	Age-standardized	0.20 (0.91, −0.38)	0.02 (0.09, −0.03)
Russian Federation	Age-standardized	0.00 (0.02, −0.01)	0.00 (0.00, 0.00)
Rwanda	Age-standardized	15.34 (59.37, −37.34)	0.64 (3.19, −1.75)
Saint Kitts and Nevis	Age-standardized	0.23 (1.19, −0.38)	0.01 (0.07, −0.02)
Saint Lucia	Age-standardized	0.14 (0.70, −0.29)	0.01 (0.03, −0.01)
Saint Vincent and the Grenadines	Age-standardized	0.38 (1.89, −0.67)	0.01 (0.08, −0.02)
Samoa	Age-standardized	1.41 (5.92, −1.56)	0.04 (0.19, −0.04)
San Marino	Age-standardized	0.00 (0.00, 0.00)	0.00 (0.00, 0.00)
Sao Tome and Principe	Age-standardized	20.19 (79.74, −50.40)	0.21 (0.94, −0.34)
Saudi Arabia	Age-standardized	0.27 (1.25, −0.36)	0.00 (0.00, 0.00)
Senegal	Age-standardized	19.58 (79.69, −71.34)	0.34 (1.55, −0.49)
Serbia	Age-standardized	0.16 (0.68, −0.21)	0.01 (0.05, −0.03)
Seychelles	Age-standardized	0.28 (1.29, −0.32)	0.01 (0.05, −0.01)
Sierra Leone	Age-standardized	35.81 (150.59, −147.64)	1.22 (7.44, −6.53)
Singapore	Age-standardized	0.00 (0.02, −0.01)	0.00 (0.00, 0.00)
Slovakia	Age-standardized	0.02 (0.09, −0.03)	0.01 (0.04, −0.01)
Slovenia	Age-standardized	0.00 (0.02, −0.01)	0.00 (0.01, 0.00)
Solomon Islands	Age-standardized	6.91 (29.62, −16.36)	0.85 (3.62, −1.30)
Somalia	Age-standardized	57.61 (237.10, −191.14)	21.72 (98.13, −69.35)
South Africa	Age-standardized	6.69 (30.53, −13.30)	0.65 (3.07, −0.93)
South Sudan	Age-standardized	36.64 (154.04, −92.01)	6.51 (33.51, −20.68)
Spain	Age-standardized	0.00 (0.01, 0.00)	0.00 (0.00, 0.00)
Sri Lanka	Age-standardized	0.53 (2.58, −1.68)	0.01 (0.04, −0.01)
Sudan	Age-standardized	17.53 (75.70, −34.47)	0.30 (1.71, −0.24)
Suriname	Age-standardized	0.67 (3.35, −1.71)	0.07 (0.35, −0.10)
Sweden	Age-standardized	0.00 (0.00, 0.00)	0.00 (0.00, 0.00)
Switzerland	Age-standardized	0.00 (0.00, 0.00)	0.00 (0.00, 0.00)
Syrian Arab Republic	Age-standardized	1.50 (6.15, −2.40)	0.01 (0.06, −0.01)
Taiwan (Province of China)	Age-standardized	0.01 (0.06, −0.02)	0.00 (0.00, 0.00)
Tajikistan	Age-standardized	3.13 (15.05, −7.62)	0.60 (2.94, −1.24)
Thailand	Age-standardized	1.64 (7.09, −2.00)	0.01 (0.06, −0.02)
Timor-Leste	Age-standardized	13.79 (71.22, −51.68)	0.46 (2.36, −1.17)
Togo	Age-standardized	18.10 (79.99, −68.27)	1.12 (5.57, −5.41)
Tokelau	Age-standardized	1.41 (6.35, −2.98)	0.61 (3.03, −1.17)
Tonga	Age-standardized	1.41 (5.70, −1.79)	0.04 (0.17, −0.04)
Trinidad and Tobago	Age-standardized	0.06 (0.30, −0.09)	0.00 (0.02, −0.01)
Tunisia	Age-standardized	0.42 (1.87, −0.37)	0.00 (0.02, 0.00)
Turkey	Age-standardized	1.91 (8.82, −3.49)	0.01 (0.05, −0.01)
Turkmenistan	Age-standardized	0.96 (4.66, −1.74)	0.01 (0.06, −0.02)
Tuvalu	Age-standardized	5.52 (22.48, −10.72)	0.26 (1.21, −0.44)
Uganda	Age-standardized	19.75 (79.30, −44.97)	0.30 (1.56, −1.30)
Ukraine	Age-standardized	0.01 (0.03, −0.01)	0.00 (0.00, 0.00)
United Arab Emirates	Age-standardized	0.07 (0.36, −0.06)	0.00 (0.01, 0.00)
United Kingdom	Age-standardized	0.00 (0.00, 0.00)	0.00 (0.00, 0.00)
United Republic of Tanzania	Age-standardized	11.95 (50.07, −29.29)	0.73 (3.15, −1.72)
United States of America	Age-standardized	0.00 (0.00, 0.00)	0.00 (0.00, 0.00)
United States Virgin Islands	Age-standardized	0.02 (0.09, −0.03)	0.00 (0.01, 0.00)
Uruguay	Age-standardized	0.17 (0.83, −0.32)	0.01 (0.06, −0.02)
Uzbekistan	Age-standardized	0.63 (3.16, −1.15)	0.00 (0.02, −0.01)
Vanuatu	Age-standardized	5.21 (20.77, −12.15)	1.11 (4.64, −2.17)
Venezuela (Bolivarian Republic of)	Age-standardized	0.80 (3.90, −1.38)	0.10 (0.50, −0.11)
Vietnam	Age-standardized	2.15 (8.75, −2.52)	0.01 (0.03, 0.00)
Yemen	Age-standardized	21.93 (92.64, −51.10)	0.54 (2.53, −0.46)
Zambia	Age-standardized	23.67 (98.79, −54.33)	1.00 (4.24, −2.32)
Zimbabwe	Age-standardized	4.76 (18.96, −9.13)	3.24 (14.00, −6.82)
YLD (Years Lived with Disability)			
Afghanistan	Age-standardized	135.86 (239.09, 57.16)	60.76 (101.98, 33.64)
Albania	Age-standardized	102.23 (205.13, 50.64)	20.12 (39.84, 9.65)
Algeria	Age-standardized	40.60 (82.17, 18.11)	14.44 (24.53, 7.83)
American Samoa	Age-standardized	26.11 (45.66, 12.71)	12.15 (22.96, 5.55)
Andorra	Age-standardized	1.44 (3.71, 0.17)	0.31 (0.80, 0.01)
Angola	Age-standardized	126.50 (237.61, 15.35)	53.73 (93.23, 28.79)
Antigua and Barbuda	Age-standardized	26.16 (50.99, 12.82)	13.37 (24.22, 7.13)
Argentina	Age-standardized	30.32 (71.60, 8.97)	8.86 (24.28, 2.90)
Armenia	Age-standardized	1.91 (3.97, 0.69)	0.70 (1.47, 0.25)
Australia	Age-standardized	0.03 (0.07, 0.01)	0.01 (0.01, 0.00)
Austria	Age-standardized	2.17 (5.79, −0.14)	0.36 (0.97, 0.03)
Azerbaijan	Age-standardized	11.87 (24.65, 4.99)	4.85 (9.20, 2.25)
Bahamas	Age-standardized	15.98 (28.41, 8.30)	11.18 (18.44, 5.85)
Bahrain	Age-standardized	10.43 (20.77, 4.55)	2.36 (4.85, 1.02)
Bangladesh	Age-standardized	94.54 (174.02, 40.13)	11.69 (20.63, 5.84)
Barbados	Age-standardized	15.91 (32.00, 7.72)	9.75 (17.76, 5.08)
Belarus	Age-standardized	2.90 (5.80, 1.20)	0.50 (1.15, 0.16)
Belgium	Age-standardized	1.95 (4.66, 0.34)	0.35 (0.85, 0.07)
Belize	Age-standardized	47.51 (88.26, 23.68)	20.74 (38.79, 10.93)
Benin	Age-standardized	215.42 (362.15, 100.25)	120.89 (205.88, 65.41)
Bermuda	Age-standardized	5.23 (11.14, 2.19)	1.20 (2.58, 0.43)
Bhutan	Age-standardized	158.05 (257.00, 89.28)	32.32 (77.66, 13.83)
Bolivia (Plurinational State of)	Age-standardized	49.87 (90.52, 22.46)	22.47 (45.12, 11.05)
Bosnia and Herzegovina	Age-standardized	51.47 (98.01, 25.16)	13.94 (26.32, 6.62)
Botswana	Age-standardized	133.69 (248.28, 57.47)	41.62 (86.65, 18.77)
Brazil	Age-standardized	66.50 (115.96, 31.57)	23.49 (40.74, 12.53)
Brunei Darussalam	Age-standardized	3.41 (7.25, 1.31)	0.71 (1.81, 0.22)
Bulgaria	Age-standardized	32.17 (64.04, 15.21)	12.02 (23.55, 5.56)
Burkina Faso	Age-standardized	344.12 (563.13, 162.56)	134.74 (230.20, 73.46)
Burundi	Age-standardized	163.10 (286.14, 69.29)	64.10 (119.81, 32.72)
Cabo Verde	Age-standardized	128.49 (256.37, 48.38)	22.43 (49.55, 10.13)
Cambodia	Age-standardized	195.39 (352.92, 90.18)	44.54 (86.77, 21.38)
Cameroon	Age-standardized	204.49 (343.63, 85.81)	66.35 (123.26, 33.20)
Canada	Age-standardized	1.07 (2.96, −0.04)	0.17 (0.38, 0.06)
Central African Republic	Age-standardized	239.72 (405.65, 107.08)	167.17 (279.03, 89.57)
Chad	Age-standardized	341.23 (586.65, 155.17)	190.30 (336.47, 100.25)
Chile	Age-standardized	9.73 (19.78, 3.36)	1.64 (3.48, 0.58)
China	Age-standardized	27.17 (45.65, 13.27)	6.15 (9.61, 3.48)
Colombia	Age-standardized	17.58 (33.69, 7.70)	4.30 (7.18, 2.18)
Comoros	Age-standardized	174.28 (360.32, 62.28)	52.57 (114.57, 24.86)
Congo	Age-standardized	203.38 (357.59, 70.03)	141.76 (251.90, 76.33)
Cook Islands	Age-standardized	43.70 (87.88, 21.03)	9.39 (17.03, 4.65)
Costa Rica	Age-standardized	12.33 (22.55, 5.88)	4.22 (7.52, 2.05)
Croatia	Age-standardized	19.32 (36.11, 9.71)	5.61 (10.60, 2.59)
Cuba	Age-standardized	10.44 (22.05, 4.66)	3.69 (7.54, 1.50)
Cyprus	Age-standardized	4.25 (9.67, 1.03)	0.50 (1.32, 0.02)
Czechia	Age-standardized	19.41 (38.04, 8.91)	4.46 (9.01, 1.89)
Cote d’Ivoire	Age-standardized	198.50 (330.34, 101.83)	78.77 (139.33, 38.98)
Democratic People’s Republic of Korea	Age-standardized	50.29 (91.00, 24.03)	16.21 (28.92, 7.40)
Democratic Republic of the Congo	Age-standardized	181.72 (318.90, 81.95)	103.41 (174.05, 56.79)
Denmark	Age-standardized	1.30 (3.22, 0.26)	0.24 (0.62, 0.02)
Djibouti	Age-standardized	132.89 (275.15, 49.36)	45.78 (94.73, 21.64)
Dominica	Age-standardized	22.53 (39.71, 12.41)	11.95 (19.00, 6.56)
Dominican Republic	Age-standardized	46.98 (84.17, 22.51)	17.76 (30.10, 9.47)
Ecuador	Age-standardized	28.67 (73.25, 3.74)	7.93 (12.96, 4.08)
Egypt	Age-standardized	32.04 (65.06, 11.19)	10.04 (16.94, 5.12)
El Salvador	Age-standardized	40.19 (80.24, 15.42)	8.95 (15.46, 4.62)
Equatorial Guinea	Age-standardized	269.02 (445.13, 121.25)	39.90 (70.62, 21.24)
Eritrea	Age-standardized	241.71 (467.10, 83.01)	80.34 (177.88, 37.33)
Estonia	Age-standardized	2.15 (4.37, 0.84)	0.27 (0.55, 0.11)
Eswatini	Age-standardized	112.96 (220.07, 39.25)	38.64 (79.07, 17.82)
Ethiopia	Age-standardized	222.55 (361.38, 106.64)	69.11 (112.68, 38.35)
Fiji	Age-standardized	50.86 (90.40, 24.87)	24.53 (54.26, 10.97)
Finland	Age-standardized	1.72 (4.01, 0.36)	0.33 (0.86, 0.04)
France	Age-standardized	0.41 (1.02, 0.06)	0.14 (0.38, 0.00)
Gabon	Age-standardized	105.54 (191.92, 51.07)	42.27 (83.66, 20.68)
Gambia	Age-standardized	268.79 (473.74, 141.23)	114.60 (226.53, 57.66)
Georgia	Age-standardized	6.25 (13.54, 2.56)	3.72 (7.75, 1.60)
Germany	Age-standardized	0.52 (1.22, 0.10)	0.13 (0.31, 0.04)
Ghana	Age-standardized	236.31 (391.76, 108.17)	99.63 (179.56, 50.33)
Greece	Age-standardized	3.03 (7.31, 0.53)	0.82 (2.01, 0.15)
Greenland	Age-standardized	0.90 (2.16, 0.24)	0.28 (0.57, 0.11)
Grenada	Age-standardized	45.13 (77.41, 23.38)	15.85 (27.64, 8.65)
Guam	Age-standardized	18.40 (35.04, 7.86)	6.27 (12.33, 2.68)
Guatemala	Age-standardized	41.55 (76.00, 16.66)	10.94 (20.16, 5.47)
Guinea	Age-standardized	247.79 (405.73, 120.93)	116.70 (204.48, 60.13)
Guinea-Bissau	Age-standardized	277.27 (511.38, 129.88)	116.79 (215.85, 58.77)
Guyana	Age-standardized	56.42 (107.15, 27.82)	18.80 (34.30, 9.93)
Haiti	Age-standardized	132.17 (243.89, 62.01)	80.89 (168.32, 38.34)
Honduras	Age-standardized	31.12 (54.72, 14.94)	9.74 (17.80, 4.89)
Hungary	Age-standardized	26.32 (50.81, 12.62)	7.05 (13.81, 3.25)
Iceland	Age-standardized	1.40 (3.62, 0.15)	0.27 (0.69, 0.02)
India	Age-standardized	171.15 (274.49, 90.31)	59.70 (98.90, 33.40)
Indonesia	Age-standardized	124.43 (216.61, 44.09)	22.77 (39.31, 11.26)
Iran (Islamic Republic of)	Age-standardized	28.25 (56.74, 11.53)	2.79 (5.33, 1.30)
Iraq	Age-standardized	46.58 (88.07, 21.54)	15.13 (25.28, 7.99)
Ireland	Age-standardized	2.02 (4.86, 0.49)	0.25 (0.67, 0.03)
Israel	Age-standardized	13.48 (28.23, 4.01)	3.30 (7.36, 0.93)
Italy	Age-standardized	2.65 (5.77, 0.93)	0.62 (1.31, 0.22)
Jamaica	Age-standardized	30.74 (53.38, 15.91)	15.55 (27.58, 8.23)
Japan	Age-standardized	0.94 (2.44, 0.16)	0.31 (0.90, −0.01)
Jordan	Age-standardized	41.90 (73.79, 21.37)	16.77 (28.29, 9.29)
Kazakhstan	Age-standardized	81.89 (175.61, 35.89)	26.87 (58.06, 11.07)
Kenya	Age-standardized	150.40 (250.42, 56.25)	69.31 (107.87, 39.13)
Kiribati	Age-standardized	139.62 (254.96, 60.00)	79.78 (146.83, 41.00)
Kuwait	Age-standardized	13.45 (22.41, 7.08)	9.51 (16.07, 4.82)
Kyrgyzstan	Age-standardized	24.83 (48.19, 12.22)	16.86 (31.03, 8.76)
Lao People’s Democratic Republic	Age-standardized	176.40 (335.50, 55.97)	40.31 (74.14, 19.43)
Latvia	Age-standardized	2.09 (4.21, 0.77)	0.35 (0.76, 0.12)
Lebanon	Age-standardized	14.89 (30.93, 6.68)	2.80 (5.77, 1.20)
Lesotho	Age-standardized	135.24 (254.67, 47.40)	58.40 (114.22, 30.30)
Liberia	Age-standardized	195.75 (339.13, 85.84)	72.06 (129.52, 37.07)
Libya	Age-standardized	27.45 (50.63, 13.79)	13.68 (22.45, 7.48)
Lithuania	Age-standardized	2.17 (4.37, 0.86)	0.27 (0.54, 0.12)
Luxembourg	Age-standardized	1.41 (3.27, 0.35)	0.22 (0.53, 0.04)
Madagascar	Age-standardized	158.56 (301.11, 51.10)	62.95 (119.78, 32.23)
Malawi	Age-standardized	253.11 (430.01, 118.46)	92.64 (179.05, 46.25)
Malaysia	Age-standardized	13.17 (23.47, 6.52)	1.33 (2.65, 0.52)
Maldives	Age-standardized	204.16 (393.86, 93.83)	15.12 (29.70, 6.99)
Mali	Age-standardized	349.35 (601.72, 156.59)	178.31 (332.10, 97.82)
Malta	Age-standardized	3.89 (8.65, 0.90)	0.70 (1.75, 0.06)
Marshall Islands	Age-standardized	144.96 (239.66, 77.42)	74.10 (140.35, 38.28)
Mauritania	Age-standardized	183.54 (357.79, 82.59)	49.12 (104.73, 22.88)
Mauritius	Age-standardized	52.41 (106.71, 21.05)	9.44 (17.95, 4.52)
Mexico	Age-standardized	34.98 (59.12, 15.07)	11.02 (17.74, 6.15)
Micronesia (Federated States of)	Age-standardized	168.12 (292.45, 89.69)	87.97 (162.49, 47.09)
Monaco	Age-standardized	0.49 (1.38, 0.01)	0.13 (0.34, 0.00)
Mongolia	Age-standardized	25.69 (42.20, 13.65)	9.45 (15.59, 5.01)
Montenegro	Age-standardized	18.96 (37.20, 9.10)	7.16 (14.92, 3.11)
Morocco	Age-standardized	76.04 (144.00, 26.71)	22.20 (38.88, 12.15)
Mozambique	Age-standardized	255.48 (431.48, 111.61)	96.15 (177.01, 50.12)
Myanmar	Age-standardized	186.68 (351.06, 66.57)	36.83 (74.48, 15.92)
Namibia	Age-standardized	91.00 (184.76, 37.85)	30.39 (60.63, 14.68)
Nauru	Age-standardized	61.62 (126.50, 30.22)	37.03 (77.50, 17.74)
Nepal	Age-standardized	127.89 (255.87, 52.65)	23.77 (52.78, 10.77)
Netherlands	Age-standardized	1.17 (2.88, 0.17)	0.28 (0.83, −0.05)
New Zealand	Age-standardized	0.69 (1.87, 0.22)	0.20 (0.63, 0.06)
Nicaragua	Age-standardized	26.04 (51.96, 7.62)	3.89 (6.58, 1.91)
Niger	Age-standardized	375.55 (646.93, 157.85)	245.18 (395.74, 142.59)
Nigeria	Age-standardized	100.68 (174.55, 44.71)	35.67 (58.11, 19.46)
Niue	Age-standardized	45.28 (90.24, 21.64)	17.03 (31.82, 8.33)
North Macedonia	Age-standardized	54.33 (103.76, 24.36)	16.01 (30.13, 7.48)
Northern Mariana Islands	Age-standardized	15.59 (31.55, 6.96)	8.79 (17.60, 3.72)
Norway	Age-standardized	1.19 (2.61, 0.41)	0.14 (0.31, 0.04)
Oman	Age-standardized	64.03 (113.66, 33.91)	18.28 (29.79, 9.86)
Pakistan	Age-standardized	132.59 (256.01, 62.37)	25.35 (44.57, 14.09)
Palau	Age-standardized	30.28 (60.42, 13.69)	14.34 (28.17, 6.91)
Palestine	Age-standardized	55.75 (101.07, 28.52)	16.58 (27.45, 8.88)
Panama	Age-standardized	13.67 (27.22, 5.41)	5.34 (9.30, 2.49)
Papua New Guinea	Age-standardized	64.60 (126.01, 27.31)	36.74 (75.46, 16.23)
Paraguay	Age-standardized	50.44 (105.30, 12.32)	16.36 (28.57, 8.51)
Peru	Age-standardized	55.89 (113.05, 18.64)	16.22 (29.50, 8.33)
Philippines	Age-standardized	60.78 (119.19, 13.63)	21.93 (39.43, 10.95)
Poland	Age-standardized	35.22 (66.27, 17.11)	6.68 (13.33, 2.88)
Portugal	Age-standardized	6.07 (13.36, 1.44)	1.05 (2.42, 0.28)
Puerto Rico	Age-standardized	7.17 (15.67, 2.96)	1.34 (3.06, 0.47)
Qatar	Age-standardized	5.73 (11.13, 2.55)	0.80 (1.68, 0.28)
Republic of Korea	Age-standardized	2.04 (4.08, 0.74)	0.13 (0.29, 0.05)
Republic of Moldova	Age-standardized	4.84 (11.10, 1.83)	1.05 (2.26, 0.35)
Romania	Age-standardized	35.88 (66.26, 18.14)	9.84 (19.20, 4.39)
Russian Federation	Age-standardized	0.63 (1.32, 0.25)	0.11 (0.20, 0.05)
Rwanda	Age-standardized	115.84 (216.93, 32.39)	34.70 (60.16, 18.31)
Saint Kitts and Nevis	Age-standardized	27.74 (53.24, 13.75)	12.53 (21.53, 6.63)
Saint Lucia	Age-standardized	33.83 (66.34, 16.67)	15.26 (27.51, 8.05)
Saint Vincent and the Grenadines	Age-standardized	40.94 (80.17, 20.18)	18.50 (33.47, 9.67)
Samoa	Age-standardized	73.56 (142.32, 34.66)	39.02 (76.75, 18.62)
San Marino	Age-standardized	0.87 (2.21, 0.13)	0.23 (0.57, 0.02)
Sao Tome and Principe	Age-standardized	198.04 (349.73, 90.91)	58.06 (119.39, 26.63)
Saudi Arabia	Age-standardized	6.09 (11.83, 2.65)	0.45 (0.93, 0.17)
Senegal	Age-standardized	278.89 (504.69, 135.29)	46.85 (100.51, 21.44)
Serbia	Age-standardized	62.43 (113.30, 32.91)	16.27 (31.34, 7.79)
Seychelles	Age-standardized	25.64 (50.88, 12.13)	6.40 (10.94, 3.33)
Sierra Leone	Age-standardized	234.43 (392.86, 93.57)	107.78 (185.29, 58.13)
Singapore	Age-standardized	2.00 (5.78, 0.63)	0.21 (0.50, 0.08)
Slovakia	Age-standardized	26.39 (49.99, 12.49)	6.34 (12.14, 2.84)
Slovenia	Age-standardized	13.67 (26.72, 6.35)	3.47 (6.92, 1.45)
Solomon Islands	Age-standardized	156.14 (276.40, 80.19)	93.02 (185.63, 47.12)
Somalia	Age-standardized	353.40 (600.90, 147.23)	282.36 (491.02, 156.47)
South Africa	Age-standardized	76.48 (145.53, 27.36)	23.88 (40.55, 12.40)
South Sudan	Age-standardized	224.65 (394.56, 94.91)	131.13 (239.15, 67.77)
Spain	Age-standardized	1.87 (4.44, 0.38)	0.37 (1.00, 0.02)
Sri Lanka	Age-standardized	64.94 (126.09, 31.19)	9.55 (17.00, 4.82)
Sudan	Age-standardized	129.42 (250.36, 47.67)	36.43 (62.71, 19.59)
Suriname	Age-standardized	44.06 (84.31, 22.08)	22.71 (41.44, 12.18)
Sweden	Age-standardized	1.17 (2.70, 0.37)	0.22 (0.51, 0.05)
Switzerland	Age-standardized	0.67 (1.86, 0.05)	0.13 (0.35, 0.01)
Syrian Arab Republic	Age-standardized	38.49 (78.32, 15.05)	10.22 (19.95, 4.93)
Taiwan (Province of China)	Age-standardized	4.71 (9.63, 1.74)	0.44 (1.08, 0.08)
Tajikistan	Age-standardized	52.04 (102.82, 21.04)	29.13 (48.89, 16.12)
Thailand	Age-standardized	55.93 (99.01, 24.92)	8.69 (15.02, 4.47)
Timor-Leste	Age-standardized	168.59 (308.66, 51.92)	61.53 (112.68, 30.59)
Togo	Age-standardized	246.85 (399.63, 113.80)	101.01 (174.51, 55.19)
Tokelau	Age-standardized	69.17 (129.38, 34.99)	22.71 (45.15, 11.02)
Tonga	Age-standardized	57.10 (101.01, 29.39)	26.30 (50.05, 12.75)
Trinidad and Tobago	Age-standardized	20.54 (38.49, 10.19)	11.12 (18.91, 5.88)
Tunisia	Age-standardized	22.77 (40.29, 10.84)	9.33 (15.44, 4.83)
Turkey	Age-standardized	41.94 (82.23, 17.92)	6.29 (11.90, 2.89)
Turkmenistan	Age-standardized	22.62 (40.92, 11.26)	13.48 (22.46, 7.58)
Tuvalu	Age-standardized	124.48 (235.09, 62.67)	48.64 (101.99, 23.56)
Uganda	Age-standardized	149.71 (266.53, 63.71)	37.66 (68.07, 19.24)
Ukraine	Age-standardized	2.86 (5.95, 1.09)	0.83 (1.69, 0.34)
United Arab Emirates	Age-standardized	7.52 (16.08, 2.95)	1.26 (2.74, 0.45)
United Kingdom	Age-standardized	2.12 (4.54, 0.76)	0.45 (1.06, 0.18)
United Republic of Tanzania	Age-standardized	149.88 (258.11, 74.48)	48.41 (93.23, 22.87)
United States of America	Age-standardized	0.44 (0.93, 0.15)	0.15 (0.32, 0.06)
United States Virgin Islands	Age-standardized	8.70 (19.72, 3.63)	1.80 (4.05, 0.68)
Uruguay	Age-standardized	20.02 (50.78, 6.29)	5.32 (12.93, 1.73)
Uzbekistan	Age-standardized	38.41 (81.38, 17.66)	17.04 (32.66, 8.36)
Vanuatu	Age-standardized	111.35 (186.04, 52.94)	71.47 (123.51, 38.45)
Venezuela (Bolivarian Republic of)	Age-standardized	17.59 (30.46, 8.59)	10.25 (18.16, 5.13)
Vietnam	Age-standardized	38.29 (74.03, 13.92)	9.62 (16.83, 4.89)
Yemen	Age-standardized	176.94 (333.16, 67.50)	69.33 (111.64, 39.88)
Zambia	Age-standardized	190.92 (349.06, 76.49)	67.63 (128.38, 33.64)
Zimbabwe	Age-standardized	101.32 (189.98, 44.57)	60.76 (101.98, 33.64)

## Data Availability

Data analysed will be made available upon making a reasonable request to the corresponding authors.

## Abbreviations

The following abbreviations are used in this manuscript:

LMICs	Low-to-middle income countries
VAD	Vitamin A deficiency
WHO	World Health Organization
DALYs	Disability-adjusted life years
YLDs	Years Lost to Disability
AAPC	Average Annual Percentage Change
ASR	Age-standardized rate
SDI	Sociodemographic index score
LDI	Lag-distributed income
CI	Confidence interval
